# First in-depth analysis of the novel Th2-type cytokines in salmonid fish reveals distinct patterns of expression and modulation but overlapping bioactivities

**DOI:** 10.18632/oncotarget.7295

**Published:** 2016-02-09

**Authors:** Tiehui Wang, Petronella Johansson, Beatriz Abós, Amy Holt, Carolina Tafalla, Youshen Jiang, Alex Wang, Qiaoqing Xu, Zhitao Qi, Wenshu Huang, Maria M. Costa, Patricia Diaz-Rosales, Jason W. Holland, Christopher J. Secombes

**Affiliations:** ^1^ Scottish Fish Immunology Research Centre, School of Biological Sciences, University of Aberdeen, Aberdeen, UK; ^2^ Centro de Investigación en Sanidad Animal (CISA-INIA), Valdeolmos (Madrid), Spain; ^3^ College of Fishery and Life Science, Shanghai Ocean University, Shanghai, China; ^4^ School of Animal Science, Yangtze University, Jingzhou, Hubei Province, China; ^5^ Central Laboratory of Biology, Chemical and Biological Engineering College, Yancheng Institute of Technology, Yancheng, Jiangsu Province, China; ^6^ Fisheries College, Jimei University, Xiamen, Fujian Province, China; ^7^ Instituto de Investigaciones Marinas, Consejo Superior de Investigaciones Científicas (CSIC), Vigo, Spain

**Keywords:** rainbow trout *Oncorhynchus mykiss*, IL-4/13A and 13B, expression, bioactivity, type-2 immunity, Immunology and Microbiology Section, Immune response, Immunity

## Abstract

IL-4 and IL-13 are closely related canonical type-2 cytokines in mammals and have overlapping bioactivities via shared receptors. They are frequently activated together as part of the same immune response and are the signature cytokines produced by T-helper (Th)2 cells and type-2 innate lymphoid cells (ILC2), mediating immunity against extracellular pathogens. Little is known about the origin of type-2 responses, and whether they were an essential component of the early adaptive immune system that gave a fitness advantage by limiting collateral damage caused by metazoan parasites. Two evolutionary related type-2 cytokines, IL-4/13A and IL-4/13B, have been identified recently in several teleost fish that likely arose by duplication of an ancestral IL-4/13 gene as a consequence of a whole genome duplication event that occurred at the base of this lineage. However, studies of their comparative expression levels are largely missing and bioactivity analysis has been limited to IL-4/13A in zebrafish. Through interrogation of the recently released salmonid genomes, species in which an additional whole genome duplication event has occurred, four genomic IL-4/13 loci have been identified leading to the cloning of three active genes, IL-4/13A, IL-4/13B1 and IL-4/13B2, in both rainbow trout and Atlantic salmon. Comparative expression analysis by real-time PCR in rainbow trout revealed that the IL-4/13A expression is broad and high constitutively but less responsive to pathogen-associated molecular patterns (PAMPs) and pathogen challenge. In contrast, the expression of IL-4/13B1 and IL-4/13B2 is low constitutively but is highly induced by viral haemorrhagic septicaemia virus (VHSH) infection and during proliferative kidney disease (PKD) *in vivo*, and by formalin-killed bacteria, PAMPs, the T cell mitogen PHA, and the T-cell cytokines IL-2 and IL-21 *in vitro*. Moreover, bioactive recombinant cytokines of both IL-4/13A and B were produced and found to have shared but also distinct bioactivities. Both cytokines rapidly induce the gene expression of antimicrobial peptides and acute phase proteins, providing an effector mechanism of fish type-2 cytokines in immunity. They are anti-inflammatory via up-regulation of IL-10 and down-regulation of IL-1β and IFN-γ. They modulate the expression of cellular markers of T cells, macrophages and B cells, the receptors of IFN-γ, the IL-6 cytokine family and their own potential receptors, suggesting multiple target cells and important roles of fish type-2 cytokines in the piscine cytokine network. Furthermore both cytokines increased the number of IgM secreting B cells but had no effects on the proliferation of IgM^+^ B cells *in vitro*. Taken as a whole, fish IL-4/13A may provide a basal level of type-2 immunity whilst IL-4/13B, when activated, provides an enhanced type-2 immunity, which may have an important role in specific cell-mediated immunity. To our knowledge this is the first in-depth analysis of the expression, modulation and bioactivities of type-2 cytokines in the same fish species, and in any early vertebrate. It contributes to a broader understanding of the evolution of type-2 immunity in vertebrates, and establishes a framework for further studies and manipulation of type-2 cytokines in fish.

## INTRODUCTION

Mammalian interleukin (IL)-4 and IL-13 were among the first cytokines described in the early 1980s [1-2]. IL-4 was initially identified as a T cell-derived B cell growth factor [3] and an immunoglobulin (Ig) switch factor for IgG1 [4] and IgE [5], and was later found to act also on T cells, mast cells [6], macrophages, cells of the myeloid and erythroid lineages, and non-hemopoietic cells [7]. In 1989, IL-13, a close congener of IL-4, was found to mediate virtually all of the IL-4 actions on non-haematopoietic cells and to some degree on haematopoietic cells [8]. Although they share low amino acid sequence identity, eg 23% in humans and 22% in cow, IL-4 and IL-13 are indeed closely related. They sit side by side in the mammalian genome and form part of a contiguous gene cluster sharing regulatory elements, and are frequently activated together as part of the same immune response [1, 9-10]. They function not only in the immune system but also in pregnancy, fetal development, mammary development and lactation, and in higher brain functions including memory and learning in mammals [11].

In the mammalian immune system, IL-4 and IL-13 are type-2 cytokines that drive canonical type-2 immunity, characterised by eosinophilia, mast cell hyperplasia, IgE secretion, smooth muscle contraction and epithelial remodelling [1, 9, 12-13]. These responses offer protection against helminth parasites but can also be damaging when activated inappropriately, leading to type-2 inflammatory conditions such as allergy and asthma [13-15]. IL-4 and IL-13 are produced by CD4^+^ T-helper type 2 (Th2) cells, effecting host immunity against extracellular parasites. The canonical type-2 cytokine-induced responses are mediated *via* STAT (Signal Transducer and Activator of Transcription)6/GATA3. The binding of IL-4 or IL-13 to their receptors results in the phosphorylation of STAT6, which dimerizes, translocates to the nucleus and induces GATA3 expression. This signaling pathway creates a positive feedback loop to maintain IL-4 and IL-13 production in Th2 cells. Thus, GATA3 promotes type-2 cytokine expression, and additionally auto-activates its own transcription thereby stabilizing the Th2 fate [16]. IL-4 and IL-13 production in Th cells can also be potentiated by non-canonical pathways, as seen with IL-2 that can drive IL-4 transcription in an IL-4R-independent manner through the phosphorylation of STAT5 [17].

In addition to production of IL-4 and IL-13 by mammalian Th2 cells, innate immune cells (eg basophils, eosinophils and mast cells) constitutively express both molecules and represent important sources of type-2 cytokines early during type-2 immunity [18-20]. These cells can rapidly release cytokines within 5-10 min, due to the presence of pre-formed type-2 cytokines in their secretory granules, and can also generate cytokines de novo following stimulation [21]. Furthermore, invariant Natural Killer T cells, a population of innate T lymphocytes, and type-2 innate lymphoid cells (ILC2) are also implicated as a major source of IL-4 and IL-13 production *in vivo* [22-23].

IL-4 and IL-13 signal through cell surface heterodimeric receptors composed of 3 possible subunits, IL-4Rα, IL-13Rα1 and the common-γ chain (γC). IL-4 signals through both the type I receptor composed of the IL-4Rα and γC, and the type II receptor composed of the IL-4Rα and IL-13Rα1, whilst IL-13 only signals through the type II receptor [1, 9]. IL-4 binds IL-4Rα with very high affinity (KD = 20-300 pM), leading to the recruitment of either γC or IL-13Rα1, that both have lower, approximately equal affinity for the IL-4:IL-4Rα complex (KD = 500 nM). Binding of IL-4 to the type I receptor complex activates JAK1/3. In contrast, IL-13 binds to IL-13Rα1 with moderate affinity (KD = 30 nM) relative to the IL-4:IL-4Rα interaction, leading to recruitment of the IL-4Rα subunit and activation of JAK1 or JAK2/TYK2. IL-13 can also bind with extraordinarily high affinity [< 10^−15^ M,] to IL-13Rα2, which is believed to be a “decoy” receptor for IL-13 owing to its lack of a cytoplasmic tail and signaling motifs [24]. The IL-4 and IL-13 receptor subunits are expressed at low levels under normal homeostatic conditions, but are influenced by hormones, cellular/oxidative stress, infection and inflammation [25]. Whilst the IL-4Rα and IL-13Rα1 chains are widely expressed at low levels on most cell types, the γC chain is primarily expressed on hematopoietic immune cells [9]. Therefore, the availability of each receptor subunit on the cell surface, and concentration of IL-4 *vs*. IL-13 in the extracellular milieu are important determinants of the signaling pathway activated within the responding cells [1, 9]. IL-4 and IL-13 can elicit overlapping but also unique biological responses. For example, in the pathogenesis of allergic asthma, IL-4 and IL-13 play an important role in tandem, with IL-4 having a pivotal role in Th2 cell proliferation, cytokine production and IgE synthesis, whilst IL-13 plays a pivotal role in the effector phase of the disease [14].

Little is known about the origin of type-2 responses, and whether they were an essential component of the early adaptive immune system that gave a fitness advantage by limiting collateral damage caused by metazoan parasites. In addition, whilst Th2 responses have been known for a long time [2] there are still many questions relating to the mechanisms that initiate and control these responses in mammals [26], which comparative studies in other vertebrates may aid. All the receptor subunits of IL-4 and IL-13 (IL-4Rα, γC, IL-13Rα1 and IL-13Rα2) exist throughout the jawed vertebrates [10], with two copies of each present in salmonids [27-29]. IL-4 and IL-13 are present in mammals and birds but clear orthologues of IL-4 or IL-13 are missing in other vertebrates [10]. The first IL-4/IL-13 related gene was identified adjacent to RAD50 in the pufferfish (*Tetraodon nigroviridis*) genome by Li and colleagues [30]. A second IL-4/IL-13 like gene was discovered in zebrafish (*Danio rerio*) at a different locus (adjacent to KIF3A) [31]. Subsequent analysis of the two loci has revealed that they most likely arose as a consequence of the third round (3R) whole genome duplication (WGD) event that happened in the teleost fish ancestor. Hence, the two genes were called IL-4/13A (adjacent to RAD50) and IL-4/13B (adjacent to KIF3A) [32]. More recently a single IL-4/13 gene was identified in the genome of the 2R bony fish, spotted gar (*Lepisosteus oculatus*) between KIF3A and RAD50 [10], and at least two IL-4/13 genes have been found between KIF3A and RAD50 in the elephant shark (*Callorhinchus milii*), a 2R cartilaginous fish [33-34]. It seems likely that a single IL-4/IL-13 gene existed in ancestral Gnathostomes, which has been duplicated in different lineages by WGD and/or tandem duplication events [10].

To date, functional analysis of IL-4/13A and IL-4/13B in fish is limited. The tetraodon IL-4/13A exhibits constitutive expression in most tissues studied, being higher in gills, brain, muscle and heart [30]. High constitutive expression of IL-4/13A has been found in salmonid mucosal tissues (gill, skin) [35]. In addition, IL-4/13A has been found to be up-regulated in rainbow trout *Oncorhynchus mykiss* epidermis 9 days post-infection with *Ichthyobodo necator* compared with uninfected fish [36]. Interestingly, a cell line (KoThL5) that expresses IL-4/13B has been established from carp (*Cyprinus carpio*) [37]. These cells also express TCR chains, and a CD4-like molecule (termed CD4-1), a phenotype similar to mammalian Th2 cells. Bioactivity analysis has only been carried out using recombinant zebrafish IL-4/13A [38-39]. Administration of IL-4/13A *in vivo* increased the numbers of peripheral blood leucocytes that express the IgZ-2 isoform after two days, or DC-SIGN after 5 days [38], and significantly upregulated B cell proliferation and antibody production [39]. The bioactivity of IL-4/13B is unknown in any fish species, and the comparative expression and modulation of both IL-4/13A and B paralogues in the same species is currently lacking.

In this study, we first identified four IL-4/13 genomic loci in the salmonid genome, that enabled the cloning of three active genes, IL-4/13A, B1 and B2. Comparative transcriptional analysis was undertaken in tissues from healthy fish, in cell lines and purified immune cells, and during parasitic, viral and bacterial infections. Bioactive recombinant proteins were generated for all the three trout IL-4/13 isoforms. Functional analysis revealed overlapping yet distinct functionalities in terms of transcriptional modulation of other immune genes and their impact on B cell populations.

## RESULTS

### Salmonids possess three active fish-specific Th2 cytokines, IL-4/13A, IL-4/13B1 and IL-4/13B2, and an IL-4/13A pseudogene

**Figure 1:**
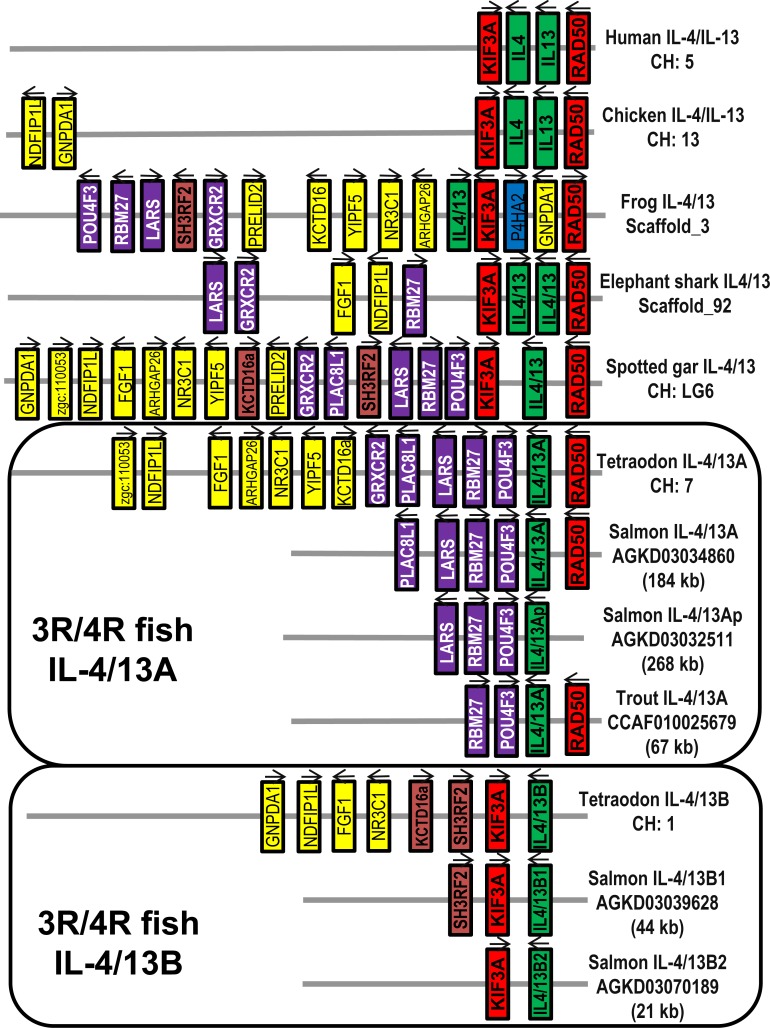
Gene synteny at the Th2 loci across vertebrates The KIF3A/IL-4/IL-13/RAD50 loci were analysed using the Genomicus program (genomicus, database release-78.01). The information for frog and elephant shark is extracted from NCBI genomic sequence NW_004668234 (frog *Xenopus (Silurana) tropicalis*) and NW_006890145 (shark *Callorhinchus milii*). The spotted gar locus was used as a reference with conserved genes colour-matched. The salmonid IL-4/13 loci were derived from the WGS contigs of Atlantic salmon and rainbow trout (Table 1).

Three IL-4/13 cDNA sequences with complete coding sequence (CDS) have been cloned in both rainbow trout and Atlantic salmon *Salmo salar* that match genomic sequences in the database (Supplementary Figures S1-S2 and 4-7, and Table 1). According to homology and linkage to RAD50 and KIF3A [1, 32] (Figure 1 and Table 1), they have been named as IL-4/13A, IL-4/13B1 and IL-4/13B2, with the likelihood that the last two genes have originated from the salmonid 4R WGD event [40]. Each gene has a potential polyadenylation signal and between 2 and 4 ATTTA motifs in the 3′-untranlated region (UTR). An in frame stop codon is also apparent in the 5′-UTR in all the genes, except salmon IL-4/13B2 (Figures S1-S2 and 4-7, and Table 1).

A Whole Genome Shotgun (WGS) sequence contig (Acc. no. AGKD03032511) was also identified in Atlantic salmon that had a putative exon corresponding to the last exon of the IL-4/13A gene (designated as IL-4/13p). This exon, when translated, has 48.7% identity to the last exon of salmon IL-4/13A and 61.5% identity to that of trout IL-4/13A, and has a potential polyadenylation signal (Figure S3). However, a canonical intron acceptor site (AG) is missing at the intron junction and ATTTA motifs are missing in the putative 3′-UTR that are present in the 3′-UTR of all the other salmonid IL-4/13 genes. No other IL-4/13 exons could be identified upstream of this last exon. Furthermore, this putative exon sits in a syntenically conserved locus, with the salmonid IL-4/13A gene downstream of genes including POU4F3, RBM27 and LARS (Figure 1). Such evidence suggests it originated from a decayed IL-4/13A gene after the salmonid 4R WGD event [40].

The salmonid IL-4/13 genes encode proteins of 142-153 aa, with predicted signal peptides of 17-24 aa, mature peptides of 119-136 aa, and 0-3 potential N-glycosylation sites (Figures S1-S2, S4-S7; Table 1). The mature peptides of salmonid IL-4/13 isoforms have theoretical molecular weights of 13.69-15.33 kDa. All trout IL-4/13 isoforms have a basic pI (7.20-7.90). Salmon IL-4/13A is also basic (pI = 8.72), but the two salmon IL-4/13B isoforms are acidic (pI = 6.72 for B1 and 4.91 for B2) (Table 1). The salmonid IL-4/13 isoforms have four conserved cysteine residues with two additional cysteine residues at the C-terminal of salmonid IL-4/13B2 (Figure S8). The aa identities of the trout and salmon molecules are highest (81.5-82.4%) between the orthologues of salmonid IL-4/13 isoforms from the 4R WGD (isoforms A, B1 or B2), although this is relatively low compared to other cytokines. Moderate identity (61.8-66.0%) is seen between the paralogues from the 4R WGD (between B1 and B2) and only low identity (26.3-29.3%) is seen between the paralogues from the 3R WGD (between A and B, Table 1).

**Table 1:** Summary of sequence analysis of salmonid IL-4/13 genes

	Features	TroutIL-4/13A	Salmon IL-4/13A	TroutIL-4/13B1	SalmonIL-4/13B1	TroutIL-4/13B2	SalmonIL-4/13B2
cDNA	Accession number	FN820500	AB574339^a^	HG794522	HG794524	HG794523	HG794525
	Length (bp)	794	455	548	696	565	683
	In frame stop codon in 5′UTR	Yes	Yes	Yes	Yes	Yes	No
	ORF (bp)	438	429	453	453	462	444
	PolyA signal	Yes	Yes	Yes	Yes	Yes	Yes
	ATTTA motifs	2	2	3	4	3	3
Genomic DNA	Accession number	FN820501	AGKD01083057	CCAF010144013	AGKD03039628	CCAF010096158	AGKD04001089
Protein	Full length (aa)	145	142	150	150	153	147
	Signal peptide (aa)	24	23	17	17	17	17
	Mature peptide (aa)	121	119	133	133	136	130
	Cysteine residues	4	4	4	4	6	6
	pI^b^	7.20	8.72	7.90	6.72	7.82	4.91
	MW^c^	13.70	13.69	15.06	15.16	15.33	14.46
	N-glycosylation sites^d^	0	2	3	2	1	2
Full-length aa identity (top right) /similarity (bottom left)^e^	Trout IL-4/13A		**81.5**^e^	29.3	28.7	26.3	28.5
	Salmon IL-4/13A	89.0		27.6	28.0	26.8	27.1
	Trout IL-4/13B1	44.7	43.3		**82.0**^e^	66.0	65.1
	Salmon IL-4/13B1	46.0	46.0	88.0		62.7	61.8
	Trout IL-4/13B2	43.8	42.5	75.8	72.5		**82.4**^e^
	Salmon IL-4/13B2	46.9	46.3	74.7	72.7	86.3	

a Salmon IL-4/13A cDNA was retrieved from the database.

b pI of predicted mature peptides.

c Theoretical molecular weight (kDa) of the predicted mature peptides.

d Potential N-glycosylation sites.

e The identities of salmonid orthologues of IL-4/13A, B1 and B2 are in bold.

### Sequence analysis reveals lineage-specific evolution

Amino acid identity/similarity analysis between fish IL-4/13 molecules, tetrapod IL-4 and IL-13, and other γ-chain cytokines revealed the salmonid IL-4/13A and B isoforms to have similar identities/similarities to other IL-4/IL-13 molecules from different lineages (Table S1). Salmonid IL-4/13 isoforms share relatively low identity (24-25%) to the predicted IL-4/13 from the spotted gar (a 2R bony fish) and the IL-4/13 molecules from (3R) cyprinids and percomorphs. Even lower identities were seen compared to homeotherm IL-4 and IL-13 (18-21%), and to other mammalian γ-chain cytokines (17-20%) (Table S1).

To see whether the salmonid molecules allow a greater insight into IL-4/13 evolution, a neighbour-joining phylogenetic tree was constructed based on a multiple alignment of fish IL-4/13, tetrapod IL-4 and IL-13, and other closely related γ-chain cytokines (IL-2, IL-9 and IL-21) (Figure S9). The salmonid IL-4/13 molecules, along with the IL-4/13 molecules from other vertebrates, and tetrapod IL-4 and IL-13 formed a large cluster with 94% bootstrap support, which was separated from other closely related γ-chain cytokines, suggesting that all the IL-4/13 genes are indeed related to tetrapod IL-4 and IL-13 as suggested by the synteny analysis (Figure 1). Lineage-specific groups, eg IL-4 and IL-13 from mammals and birds, and IL-4/13A and IL-4/13B from salmonids, cyprinids and percomorphs, are well supported. However, fish orthologue-specific groupings, as seen in phylogenetic trees of IL-1β [41], TNFα [42] and the sub-units of IL-12 [43] are missing (Figure S9). The topology of the phylogenetic tree is in agreement with the homology analysis (Table S1) in which aa identities within orthologues (eg IL-4/13A) are no higher than between paralogues (eg IL-4/13A and B), suggesting fast-evolution of the IL-4/13 related genes in vertebrates.

**Figure 2:**
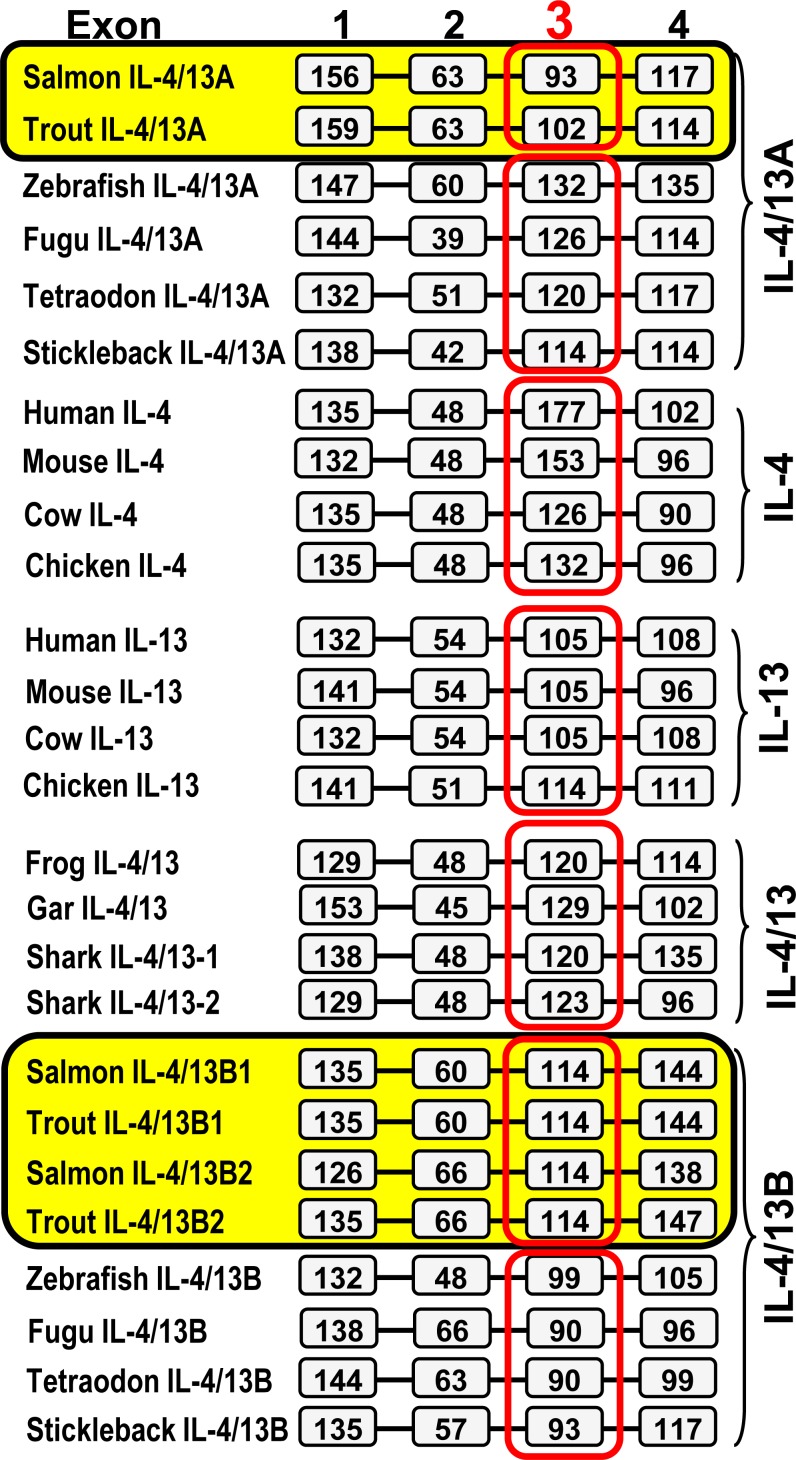
Comparison of the gene organisation of salmonid IL-4/13 genes with IL-4, IL-13 and related genes from other vertebrates The boxes represent amino acid coding regions and the black bars represent introns. The sizes (bp) of the coding regions are numbered in the boxes. The gene organisation of the salmonid IL-4/13 genes was predicted using the Spidey program based on the sequence information from Tables 1 and Figures S1-S2 and S4-S7. Other genes were extracted from NCBI genomic sequences NW_004668234 (frog IL-4/13) and NW_006890145 (elephant shark IL-4/13-1 and -2) and spotted gar chromosome LG6 (gar IL-4/13). Sequence data representing all other genes was reported by Ohtani et al. [32].

To study further the apparent rapid evolution of the vertebrate IL-4/13 related genes, we analysed their gene organisation. Homeotherm IL-4 and IL-13, the 3R and 4R bony fish IL-4/13A and IL-4/13B, and the 2R vertebrate (shark, gar, amphibian) IL-4/13 genes all have a four coding exon/three phase 0 intron organisation (Figure 2). However, exon 3 appears to be lineage-specific. Exon 3 is larger in tetrapod IL-4 (126-177 bp) than in IL-13 (105-114 bp), with exon 3 of IL-4/13 genes in 2R vertebrates being of intermediate size (120-129 bp). Salmonid IL-4/13A genes, in contrast to 3R fish species, possess a smaller exon 3. Exon 3 of human IL-4 encodes for helices B and C of the mature peptide, with helix C having an important role in IL-4 binding to its high affinity receptor chain, IL-4Rα [45-46]. Thus, lineage-specific exon 3 may contribute to the lineage specific diversification of IL-4/13 genes and may have had an impact on the functional role of IL-4/13A and IL-4/13B in different lineages/species.

**Figure 3:**
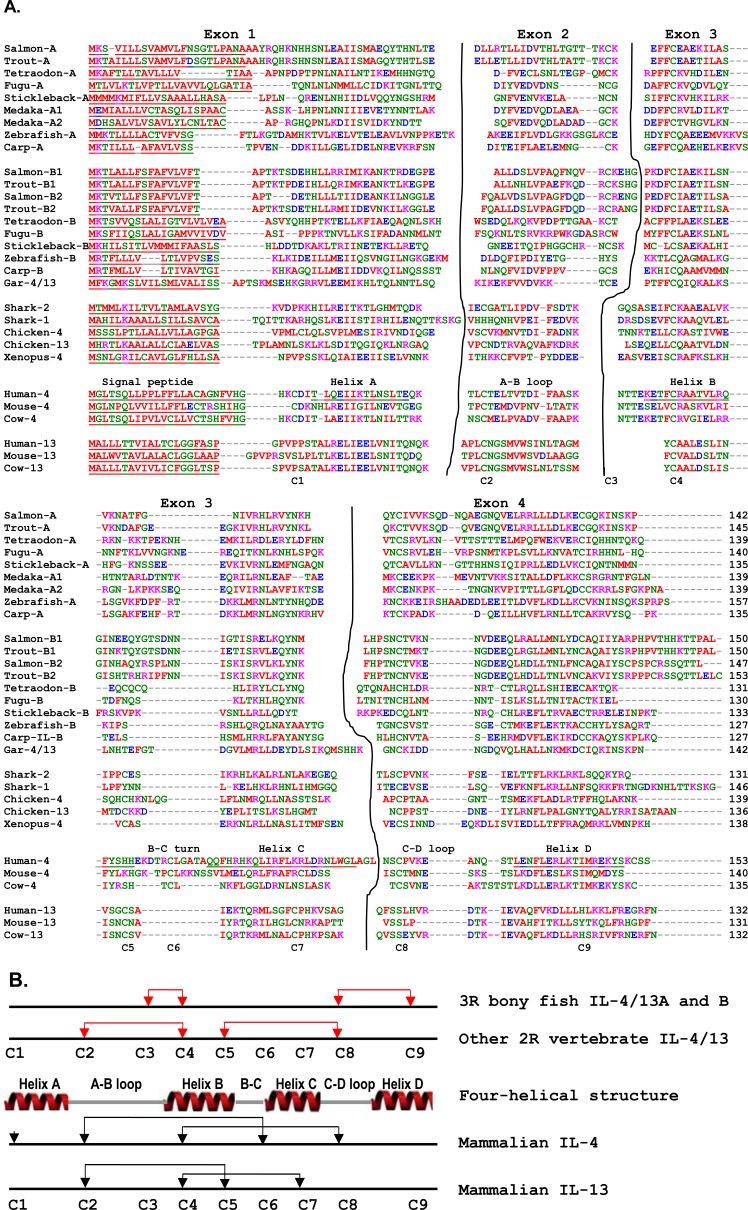
Multiple alignment of mammalian IL-4 and IL-13, and IL-4/13 related molecules from 3R bony fish and other vertebrates **A** and the patterns of cysteine residues and connectivity prediction in different vertebrate groups **A.** The exon-specific multiple alignment was produced using ClustalW. The accession numbers of the amino acid sequences used are as in Figure 2. The four exons are separated and marked above the alignment. The signal peptide, four α-helices and loops known in human IL-4 are marked above human IL-4. The nine cysteine residues conserved in different lineages are denoted under the alignment. **B.** The lineage specific conserved cysteine residues are schematically shown relative to the four helical structure of human IL-4. The known disulfide-bonds [47-48] in human IL-4 and IL-13 are shown by black lines and the predicted disulfide bonds [49] are indicated by red-lines.

To gain further insights into potential functional diversification of IL-4/13 molecules, a multiple alignment based on exon and secondary structure was generated. In human IL-4 exon 1 encodes for the signal peptide and helix A, whilst exon 2 encodes the A-B loop, exon 3 helices B and C, and the B-C turn, and exon 4 the C-D loop and helix D [46] (Figure 3A). Nine cysteine residues can be identified in the multiple alignment that are conserved in a lineage-specific manner (Figure 3B). Human IL-4 and IL-13 are known to be four-helical cytokines stabilised by two conserved disulfide bonds [47-48]. Predictions of cysteine connectivities using DISULFIND [49] in IL-4/13 molecules from fish and other vertebrates (Figure S10) suggest that lineage/species-specific disulfide bonds may have evolved to stabilise IL-4/13 molecules (Figure 3B).

### Comparative transcript expression analysis of the three trout Th2 cytokines

**Figure 4:**
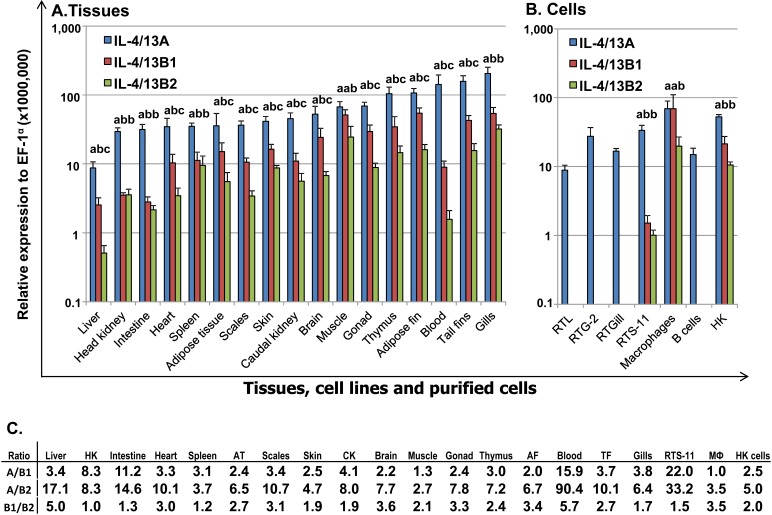
Constitutive expression of trout IL-4/13A, B1 and B2 *in vivo* in tissues **A**, in cell lines and purified immune cells **B** and the ratios of expression levels between each other **A.** The transcript expression level of the three trout IL-4/13 paralogues was determined by real time RT-PCR in 17 tissues from six fish. The transcript level was calculated using a serial dilution of references that contained equal molar amounts of the probes for each gene and was normalized against the expression level of EF-1α. The results represent the average +SEM of six fish. **B.** Total RNA was prepared from cell lines, 4-day old primary HK macrophages, FACS sorted spleen IgM^+^ B cells, and HK cells after 4 h culture *in vitro*. The expression analysis was as described in **A.**. The results represent the average + SEM from four flasks of cells or fish. A paired sample *t*-test was applied to the tissue samples, macrophages and HK cells, and one-way ANOVA was used for the cell lines. The expression levels between IL-4/13A, B1 and B2 in the same tissues or cell lines are significantly different (*p* < 0.05) when the letters over the bars are different. **C.** The ratio of the expression levels between each of the IL-4/13 paralogues. HK = head kidney, AT = adipose tissue, CK = caudal kidney, AF = adipose fin; TF = tail fins, MФ = primary macrophages.

The constitutive expression of the three trout Th2 cytokines was comparatively studied in tissues of apparently healthy fish, in cell lines, head kidney (HK) cells, primary HK macrophages, and purified IgM^+^ B cells (Figure 4). The expression of all three isoforms was detectable by real-time RT-PCR in all 17 tissues examined, with the expression of IL-4/13B2 in liver tissue being the lowest. The apparently highest expression of IL-4/13A was found in certain immune tissues/organs (gills, blood and thymus), as well as in tail fins and adipose fin. The apparently highest expression of IL-4/13B1 was also in gills and thymus, as well as in tail fins, adipose fin, muscle, gonad and brain (Figure 4A). The expression of IL-4/13A was significantly higher (*p* < 0.05) than that of IL-4/13B1 and IL-4/13B2 in all tissues except muscle where the expression of IL-4/13A and IL-4/13B1 was not significantly different (Figure 4A, 4C). The expression of IL-4/13B1 was also significantly higher (*p* < 0.05) than that of IL-4/13B2 in all tissues with the exception of gills, spleen, HK and intestine, important immune tissues/organs in fish (Figure 4A, 4C). It is noteworthy that the expression of the IL-4/13B paralogues was particularly low in immune relevant tissues such as HK, blood and intestine relative to IL-4/13A (Figure 4A).

In agreement with the high level expression in all the tissues examined, IL-4/13A expression was also detectable in all four cell lines, primary macrophages, HK cells and purified IgM^+^ B cells (Figure 4B). However, the expression of IL-4/13B1 and B2 was not detectable in RTL, RTG-2, RTGill cells or purified B cells, but was detectable in HK cells, primary macrophages and the macrophage-like cell line RTS-11. The expression of IL-4/13A was significantly higher (*p* < 0.05) than that of IL-4/13B1 and IL-4/13B2 in RTS-11 cells and in HK cells, and IL-4/13B2 in primary macrophages (Figure 4B, 4C).

### The kinetics of expression of the three trout Th2 cytokines after viral haemorrhagic septicaemia virus (VHSV) infection

**Figure 5:**
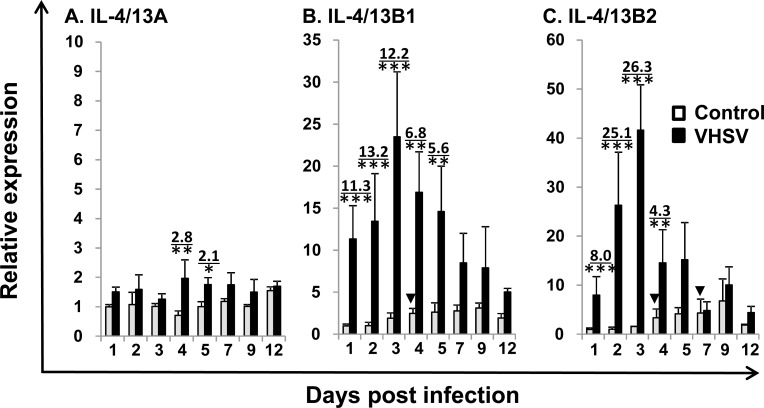
Modulation of the expression of trout IL-4/13A, B1 and B2 in kidney by VHSV infection Rainbow trout were infected by intraperitoneal (i.p.) injection with 100 μl of VHSV (DK-F1, 1 × 10^9^ TCID_50_ /ml) while the control fish were mock-infected with the same amount of control media. Fish were sampled at 1 2, 3, 4, 5, 7, 9 and 12 days post challenge, when the kidney was removed for RNA preparation and real-time RT-PCR as described in Figure 4. The relative expression of IL-4/13 paralogues was normalized to the expression of EF-1α, and expressed as arbitrary units where one unit equals the average expression level of the mock-infected fish at day 1 post infection. The results represent the mean + SEM of four fish. The relative significance of a LSD post hoc test after a significant one way-ANOVA between the VHSV and mock-injected groups at the same time point is shown above the bars as: *p < 0.05, **p < 0.01 and ***p < 0.001. The numbers above the bars are the fold changes, calculated as the average expression level of VHSV infected fish divided by that of the time-matched controls. The arrow heads indicate significant increases (*p < 0.05) in the control fish relative to control fish at day 1.

Viral haemorrhagic septicaemia is one of the most important viral diseases of salmonid fish in European aquaculture caused by VHSV [50]. To gain an insight into their role in the host immune response to pathogens, we first investigated gene expression during VHSV infection. IL-4/13A expression did not exhibit any significant changes in the control fish, however the expression of IL-4/13B1 and B2 increased over time in control fish with a significant increase at day 4 for IL-4/13B1 and at days 4 and 7 for IL-4/14B2 (Figure 5), suggesting that operational stress could modulate IL-4/13B1 and B2 expression to some extent. VHSV exposure resulted in a significant increase in IL-4/13B1 and B2 expression in the kidney during the early stages post infection (eg 1 to 5 days for IL-4/13B1, and 1-4 days for IL-4/13B2) that peaked at day 3 with a 26.3-fold increase for IL-4/13B2. IL-4/13A expression also increased but to a lesser extent (up to 2.8-fold) and only at days 4 and 5 (Figure 5).

### The expression of the three trout Th2 cytokines during proliferative kidney disease (PKD)

**Figure 6:**
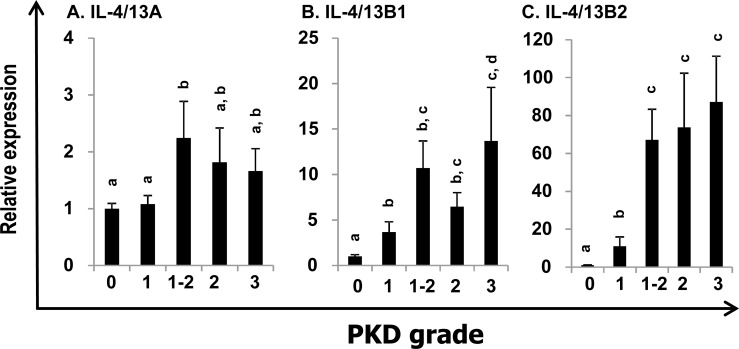
Modulation of the expression of trout IL-4/13A, B1 and B2 in kidney by parasite infection Kidneys from rainbow trout infected with *T. bryosalmonae* or from unexposed (control) fish were collected during a natural infection [51-52]. The relative expression of IL-4/13 paralogues was normalized to the expression of EF-1α, and expressed as arbitrary units where one unit equals the average expression level of the control fish. Results are presented as means + SEM. The numbers of fish analysed were 12, 5, 9, 10 and 8 representing control, grade 1, 1-2, 2, and 3, respectively. The expression levels between different groups are statistically significant (*p* < 0.05) where letters over the bars are different, as tested by one way-ANOVA.

PKD is a parasitic disease of salmonid fish caused by the myxozoan parasite *Tetracapsuloides bryosalmonae* [51]. The expression profiles of trout IL-4/13 paralogues were also investigated in the kidney of fish with clinical PKD relative to uninfected controls. The infected fish were sampled from a natural outbreak and the severity of kidney clinical pathology was assigned a swelling grade of 1-3 [51]. The expression of IL-4/13B1 and B2 was upregulated at grade 1, relative to control (grade 0) fish and to a greater extent in fish with advanced disease pathology (grades 1-2, 2 and 3) up to 13.7-fold (IL-4/13B1) or 87.2-fold (IL-4/13B2) (Figure 6B and 6C). In contrast, IL-4/13A expression was only increased 2.2-fold in grade 1-2 fish (Figure 6A).

### Modulation of the expression of the three trout Th2 cytokines *in vivo* by vaccination and bacterial challenge

**Figure 7:**
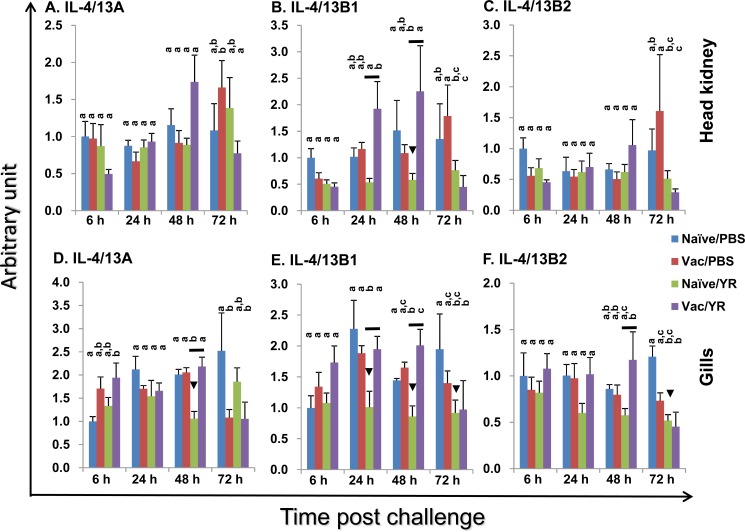
Modulation of the expression of rainbow trout IL-4/13A, B1 and B2 in head kidney **A**-**C** and gills (D-F) after vaccination and bacterial challenge A group of fish was i.p. injected with AquaVac^TM^ ERM (Vac), with naïve fish (Naïve) serving as control. The fish were challenged 60 days later by i.p. injection of *Y. ruckeri* (0.5 ml/fish, 1×10^6^ cfu/ml; YR) or injected with 0.5 ml of PBS as control (PBS). The fish from each group were killed and the gills and HK collected for total RNA extraction. The gene expression analysis was performed as in Figure 4. The expression level was expressed as arbitrary units where one unit equals the average expression level in the PBS injected control group at 6 h in each tissue. The means + SEM of five fish are shown. The expression levels between different groups of the same tissue and time point are statistically different (*p* < 0.05) where letters over the bars are different, as determined by one way-ANOVA. The down arrow head indicates a significant down-regulation in the naïve fish after bacterial challenge at the same time point. The black line over the bars indicates a significant difference between the vaccinated and naïve fish after bacterial challenge.

Enteric redmouth disease (ERM) is a serious septicaemic bacterial disease of salmonid fish species caused by *Yersinia ruckeri*, a Gram-negative rod-shaped enterobacterium. It can be prevented by bacterin vaccination [53]. Mammalian IL-4 and IL-13 are key adaptive cytokines. Thus, we examined the expression of trout IL-4/13A, B1 and B2 *in vivo* after bacterial challenge using an effective bacterial vaccination model in fish. The ERM vaccine protects the vaccinated fish from challenge and modulates the expression of adaptive cytokines involved in Th1 [53] and Th17 [54] type responses. An i.p. vaccination was performed as this is known to give a high degree of protection in the host, and when combined with an i.p. challenge means there is a clear contrast between control and vaccinated fish in terms of expecting them to live or die. In this way individual sample variation is reduced. Two important immune tissues, the HK and gills were used for gene expression analysis. The gills are an important site for ERM infection [53] and show high expression of all of the IL-4/13 paralogues, as shown in Figure 4 and by others [35]. The HK is the central immune organ of fish. Naïve fish started to show disease symptoms and to die from day 3 post bacterial infection. Thus, we focussed on early times post-infection. Gene transcriptional levels were expressed as arbitrary units where one unit in each tissue equals the average expression level in the PBS injected control group at 6 h (Figure 7). In naïve control fish, *Y. ruckeri* challenge resulted in a down-regulated expression in the gills of IL-4/13A (at 48 h), IL-4/13B1 (at 24 h, 48 h and 72 h) and IL-4/13B2 (at 72 h), and in the HK of IL-4/13B1 at 48 h post challenge (Figure 7). In vaccinated fish, *Y. ruckeri* challenge did not alter the expression of IL-4/13A, B1 and B2 in both tissues examined at early stages (6 h, 24 h and 48 h). However at 72 h, the expression of IL-4/13B1 and B2 in gills and HK, and of IL-4/13A in HK, was lower in *Y. ruckeri* challenged vaccinated fish compared to vaccinated and PBS injected fish (Figure 7). Interestingly, the expression of IL-4/13B1 was higher in vaccinated fish than in naïve fish during the early infection phase (24 h and 48 h) in both gills and HK. Similarly, the expression of IL-4/13A and IL-4/13B2 was also higher in gills at 48 h post-challenge (Figure 7).

### Modulation of the expression of the three trout Th2 cytokines in primary macrophages and HK cells

**Figure 8:**
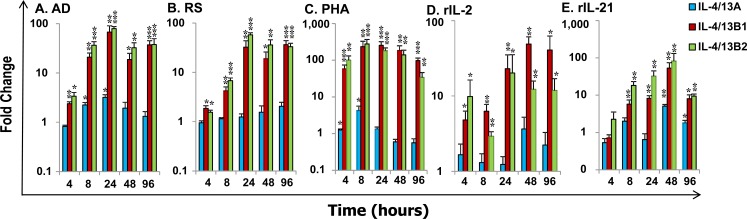
Modulation of the expression of rainbow trout IL-4/13A, B1 and B2 in HK cells Freshly prepared HK cells were stimulated with formalin killed *A. davidanieli* (AD, A) and *R. salmoninarum* (RS, B), a T cell mitogen PHA (**C**), or the recombinant trout cytokines rIL-2 (**D**) and rIL-21 (**E**) for 4 h, 8 h 24 h, 48 h and 96 h. Quantification of gene expression was as described in Figure 4. Modulated expression was expressed as a fold change calculated as the mean expression levels in stimulated cells normalized to that of time-matched controls. The means + SEM of cells from four fish are shown. The relative significance of paired sample T tests between stimulated and time-matched control samples is shown above the bars. * *p* **≤** 0.05, **p **≤** 0.01 and ***p **≤** 0.001.

To gain a further insight into IL-4/13 expression and modulation, we performed an expression analysis *in vitro* using HK cells and primary macrophages that are known to express all the three IL-4/13 paralogues (Figure 4). Bacterial infection had a negative effect on the expression of IL-4/13 paralogues *in vivo* (Figure 7). We, therefore, anticipated that host immune cells, such as HK cells that are a mixed population containing T cells, B cells, macrophages, neutrophils and others, should be modulated to express IL-4/13 paralogues in response to bacterial PAMPs (pathogen-associated molecular patterns). Thus HK cells were stimulated with formalin killed *Renibacterium salmoninarum* (a Gram positive bacterium that is the aetiological agent of bacterial kidney disease), *Y. ruckeri* and *Aeromonas salmonicida* (Gram negative bacteria, the latter being the aetiological agent of furunculosis), as well as with a non-pathogenic bacterium *Arthrobacter davidanieli* (used as a live vaccine for *R. salmoninarum*) [55]. *A. davidanieli* stimulation of HK cells markedly induced the expression of IL-4/13B paralogues from 4 h to 96 h, that peaked at 24 h with an increase of 53.5-fold for IL-4/13B1 and 78.3-fold for IL-4/13B2 (Figure 8A). It also induced the expression of IL-4/13A at 8 h and 24 h but to a lesser extent (up to 3.3-fold). Similarly pathogenic *R. salmoninarum* (Figure 8B), *Y. ruckeri* and *A. salmonicida* (data not shown) all highly induced the expression of IL-4/13B paralogues but had little to no effect on the expression of IL-4/13A (Figure 8B).

Due to the lack of reagents to isolate T cells in fish, the T-cell expressing potential of IL-4/13 paralogues was investigated in HK cells by stimulation with a T cell mitogen, phytohaemagglutinin (PHA), and two trout recombinant (r) cytokines, rIL-2 [56] and rIL-21 [57], that have major effects on T cells. PHA is a potent inducer of IL-4/13 paralogues in HK cells (Figure 8C) as well as spleen leucocytes. PHA stimulation markedly increased the expression of IL-4/13B paralogues from 4 h (53.0-fold for IL-4/13B1 and 91.0-fold for IL-4/13B2) to 96 h that peaked at 8 h for IL-4/13B2 (236.7-fold) and 24 h for IL-4/14B1 (222.3-fold). PHA also increased the expression of IL-4/13A at 4 h and 8 h, but only up to 4-fold. Recombinant rIL-2 increased the expression of IL-4B paralogues from 4 h to 96 h, with a peak of induction at 24 h for IL-4/13B2 (20.2-fold) and at 48 h for IL-4/13B1 (49.0-fold), but had no effects on IL-4/13A expression (Figure 8D). rIL-21 also increased the expression of IL-4/13B paralogues, however the effect was relatively late, from 8 h to 96 h, and peaked at 48 h with a 52.8 fold increase for IL-4/13B1 and 82.8 fold for IL-4/13B2. In contrast to the effect of rIL-2, rIL-21 also increased IL-4/13A expression at a late stage (48 h and 96 h) but with a maximal 5.1-fold increase (Figure 8E). The induction of the expression of IL-4/13B paralogues by PHA, rIL-2 and rIL-21 was observed in at least three independent experiments.

**Figure 9:**
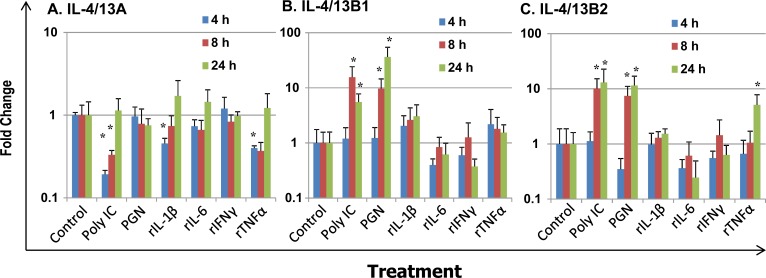
Modulation of the expression of rainbow trout IL-4/13A **A**, B1 **B** and B2 **C** in primary HK macrophages Four day old primary HK macrophages were stimulated with the PAMPs poly IC and peptidoglycan (PGN), or the recombinant cytokines rIL-1β, rIL-6, rIFN-γ and rTNFα for 4 h, 8 h and 24 h. Quantification of gene expression was as described in Figure 4. Modulated expression was expressed as a fold change calculated as the mean expression levels in stimulated cells normalized to that of time-matched controls. The means + SEM of cells from four fish are shown. The relative significance of paired sample T tests between stimulated and time-matched control samples is shown above the bars. *p **≤** 0.05.

We further investigated the modulation of expression of IL-4/13 paralogues in HK macrophages. As potential antigen presenting cells and sensors of inflammatory signals from innate and adaptive immune responses in fish, primary trout HK macrophages were stimulated with PAMPs, including polyinosinic-polycytidylic acid (poly IC, a mimic of viral infection) and peptidoglycan (PGN, major cell wall components of bacteria), and the inflammatory trout cytokines rIL-1β [58], rIL-6 [59], rIFNγ [29] and rTNFα [42] (Figure 9). IL-4/13A expression in primary macrophages was down-regulated at early time points by poly IC, rIL-1β and rTNFα at 4 h, and by poly IC at 8 h, but was refractory to stimulation with PGN, rIL-6 and rIFNγ from 4 h to 24 h (Figure 9A). The expression of both IL-4/13B paralogues was refractory at 4 h, but markedly up-regulated at 8 h and 24 h by the PAMPs. IL-4/13B1 expression was increased up to 15.6-fold by poly IC and 36.5-fold by PGN (Figure 9B). Similarly, IL-4/13B2 expression was increased up to 13.0-fold by poly IC and 11.5 fold by PGN (Figure 9C). However, the expression of the IL-4/13B paralogues in HK macrophages was refractory to stimulation with all the proinflammatory cytokines tested, with the exception of rTNFα that up-regulated IL-4/13B2 expression at 24 h (5.4-fold). The bioactivity of these cytokines have been reported previously [29, 42, 58, 59] and are confirmed in this study by the modulation of a number of marker genes.

### Production and purification of bioactive recombinant trout Th2 cytokines

**Figure 10:**
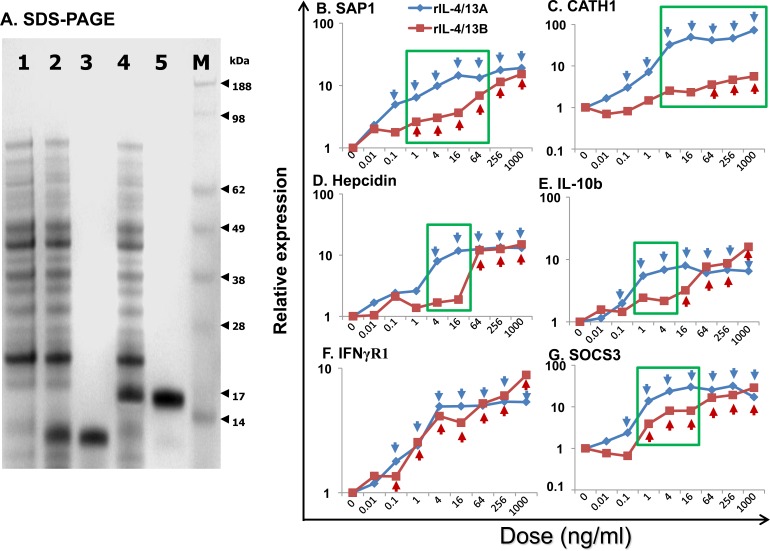
SDS-PAGE analysis **A** and dose responses **B**-**G** of trout rIL-4/13A and rIL-4/13B2 **A.** SDS-PAGE analysis of rIL-4/13A and B expressed and purified from *E. coli* BL21 Star (DE3), and stained with SeeBlue (Invitrogen). 1, a sample from un-induced BL21 cells, 2 and 4, BL21 transformed by IL-4/13A- and IL-4/13B2-expressing plasmid, respectively and induced with 1 mM IPTG for 4 h; 3 and 5, expression product purified from transformed cells expressing IL-4/13A and IL-4/13B2, respectively; and 6, Protein marker, SeeBlue Plus2 (Invitrogen). **B.**-**G.** Freshly prepared HK cells were stimulated with rIL-4/13A or rIL-4/13B2 at 0.01, 0.1, 1, 4, 16, 64, 256, and 1000 ng/ml for 4 h. Gene expression was determined as described in Figure 4. The relative gene expression was calculated as the expression levels in stimulated cells normalized to that of un-stimulated controls. The means of cells from four fish are shown. For clarity the SEM bars are not shown here but are provided in Figure S11. The blue arrows and red arrows indicate significant differences (*p* < 0.05, paired samples T tests) after stimulation with rIL-4/13A and rIL-4/13B2, respectively. A green box indicates significantly different expression levels (*p* < 0.05, paired samples T tests) between HK cells stimulated with the same doses of rIL-4/13A or rIL-4/13B2.

Proteins of the expected size of 15.2 kDa (recombinant (r)IL-4/13A), 16.5 kDa (rIL-4/13B1 (data not shown) and 16.8 kDa (rIL-4/13B2) were induced by IPTG stimulation of transformed BL21 cells, and purified under denaturing conditions with extensive washing in 1% Triton X-100 buffer to remove LPS (Figure 10A). The purified protein was refolded *in vitro* and re-purified under native conditions, and denaturants and other contaminants were removed by extensive washing of the purification column. When the refolded proteins (up to 1000 ng/ml) were added to the macrophage cell line RTS-11, no up-regulation of IL-1β and TNF-α expression (known LPS responsive genes in this system) was seen, confirming that LPS contamination in the recombinant preparations was negligible.

Initial analysis of bioactivity at 200 ng/ml in HK cells or splenocytes suggested that all the three recombinant proteins, rIL-4/13A, B1 and B2, were active in both cell populations in terms of modulation of the expression of several genes eg IL-4Rα1 and cathelicidin-1 (CATH1). Due to the ease of preparation of large numbers of cells for *in vitro* stimulation, HK cells were chosen for further dose-response and time-course experiments. Also, since the IL-4/14B2 gene was more highly induced *in vivo* and *in vitro* (Figures 5, 6 and 8), and rIL-4/13B2 was predicated to be more stable than rIL-4/13B1 (with an instability index computed to be 37.8 for rIL-4/13B2 and 46.1 for rIL-4/13B1 [60]), and was found to be easier to refold, only trout rIL-4/13A and rIL-4/13B2 were taken further for bioactivity analysis.

A dose-response analysis of gene expression in HK cells suggested that both rIL-4/13A and B2 were bioactive at or above 0.1 ng/ml, with an increased response seen with increasing doses (Figure 10B-10G). Whilst the ability of rIL-4/13A and B2 to modulate gene expression of some genes, eg IFNγR1 ([61], Figure 10F) were not distinguishable, for other genes, eg CATH1 (Figure 10C), IL-4/13A was the more potent stimulant. However, for most of the genes analysed, eg hepcidin, IL-10b, serum amyloid P (SAP)1 and SOCS3 (Figure 10B, 10D, 10E, 10G), the difference seen related to whether modulation of gene expression was found at lower doses (eg 1-64 ng/ml) or at higher doses (eg 64-256 ng/ml) with both proteins inducing similar responses.

### Time dependent modulation of transcript expression by rIL-4/13A and rIL-4/13B2 in HK cells

**Figure 11:**
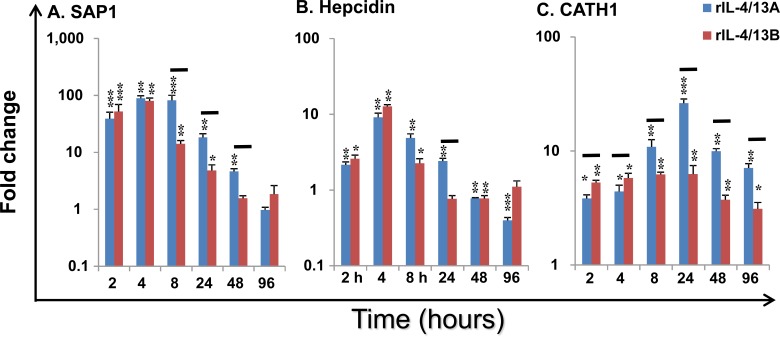
Modulation of the expression of SAP1 **A**, Hepcidin **B** and CATH1 **C** by rIL-4/13 isoforms Freshly prepared HK cells were stimulated with rIL-4/13A or rIL-4/13B2 for 2 h to 96 h and gene expression determined as described in Figure 4. Modulated expression was expressed as a fold change, calculated as the expression levels in stimulated cells normalized to that of time-matched controls. The means + SEM of cells from four fish are shown. The relative significance of paired sample T tests between stimulated and time-matched control cell cultures are shown above the bars as: *p **≤** 0.05, **p **≤** 0.01 and ***p **≤** 0.001. The black lines over the bars indicate significantly different expression levels between HK cells stimulated with rIL-4/13A and rIL-4/13B2.

**Figure 12:**
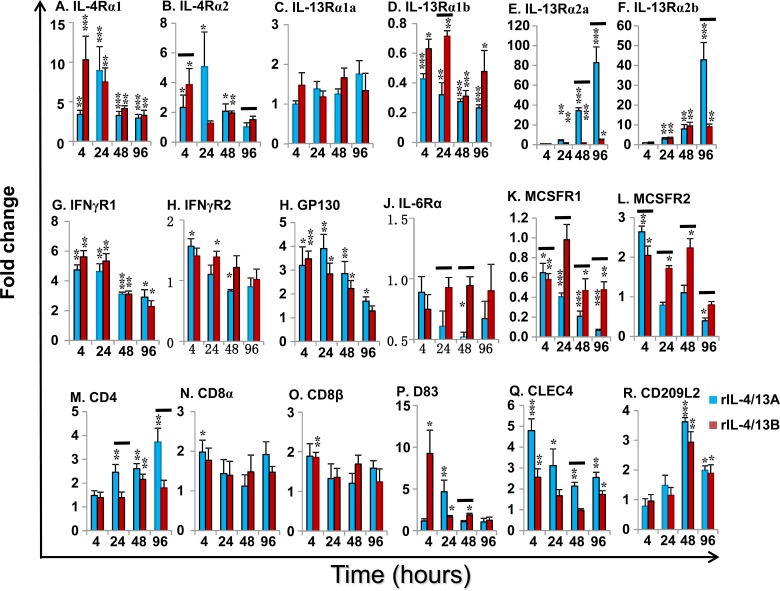
Modulation of the expression of the cytokine receptors for IL-4/13 (IL-4α1 and α2, IL-13Rα1a and α1b, and IL-13Rα2a and α2b), IFNγ (IFNγR1 and 2), IL-6 (IL-6Rα and GP130) and MCSF (MCSFR1 and 2), and cellular markers for T cells (CD4-1, CD8α and CD8β) and dendritic cells (CD83, CLEC4, and CD209L2) by rIL-4/13 isoforms Freshly prepared HK cells were stimulated with rIL-4/13A or rIL-4/13B2 for 4 h to 96 h and gene expression determined as described in Figure 11. The means + SEM of cells from four fish are shown. The relative significance of paired sample T tests between stimulated and time-matched control cell cultures are shown above the bars as: *p **≤** 0.05, **p **≤** 0.01 and ***p **≤** 0.001. The black lines over the bars indicate significantly different expression levels between HK cells stimulated with recombinant rIL-4/13A and rIL-4/13B2.

**Figure 13:**
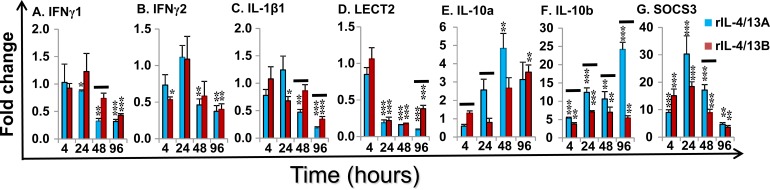
Modulation of the expression of the pro-inflammatory cytokines (IFNγ1 and 2, IL-1β1 and LECT2), anti-inflammatory cytokines (IL-10a and b) and negative regulators (SOCS3) by rIL-4/13 isoforms Freshly prepared HK cells were stimulated with rIL-4/13A or rIL-4/13B2 for 4 h to 96 h and gene expression determined as described in Figure 11. The means + SEM of cells from four fish are shown. The relative significance of paired sample T tests between stimulated and time-matched control cell cultures are shown above the bars as: *p **≤** 0.05, **p **≤** 0.01 and ***p **≤** 0.001. The black lines over the bars indicate significantly different expression levels between HK cells stimulated with recombinant rIL-4/13A and rIL-4/13B2.

A dose of 200 ng/ml was chosen for the time-course stimulation of HK cells for 4 h, 24 h, 48 h and 96 h, as both IL-4/13A and B2 had similar abilities to modulate the expression of the majority of genes examined in the dose response experiment at this concentration (Figure 10B-10G). The expression of 64 genes were analysed in detail (Figures 11, 12, 13 and Table 2) in the time dependent experiment after an initial screening of over 200 immune genes. Two additional early time points (2 h and 8 h) were also analysed for several early response genes (Figure 11).

Acute phase protein (APP) and antimicrobial peptide (AMP) genes: SAP1 expression was rapidly induced by rIL-4/13A with a 39.2-fold increase at 2 h, reaching the highest increase of 89.2-fold at 4 h and returning to control levels by 96 h. Its expression was also rapidly induced by rIL-4/13B2 with a 52.3-fold increase at 2 h, a peak of 80.4-fold at 4 h and returning to control levels by 48 h (Figure 11A). SAP2 expression was also increased but to a lesser extent, whilst another APP (serum amyloid A) was down-regulated (Table 2).

Hepcidin expression was also rapidly induced by both rIL-4/13A and B2 and peaked at 4 h but returned to (or even below) control levels at 24-96 h (Figure 11B). CATH1 expression was increased at all time points and peaked at 24 h with a 26.5-fold increase for rIL-4/13A and a 6.5-fold increase for rIL-4/13B2 (Figure 11C). However, CATH2, that has a lower constitutive expression level, was down-regulated (Table 3). It is clear that the ability to modulate APP and AMP gene expression by rIL-4/13A and B2 differ in a gene- and time-dependent manner (Figure 11).

**Table 2:** Fold change of transcript expression after stimulation of HK cells with rIL-4/13A and rIL-4/13B2

Time (h)	4 h		24 h		48 h		96 h	
rIL-4/13	A	B2	A	B2	A	B2	A	B2
Acute phase proteins and antimicrobial peptide genes								
SAP2	**0.76**	**1.01**	**1.34**	**1.41**	**1.29**	**1.36**	**1.53**	**1.92**
SAA	**0.21**	**0.77**	**1.10**	**0.71**	**0.60**	**0.59**	**0.28**	**0.79**
CATH2	**0.15**	**0.51**	**0.17**	**0.23**	**0.03**	**0.07**	**0.04**	**0.71**
Cellular marker genes								
CD4-2A	**0.88**	**1.04**	**1.25**	**1.16**	**0.93**	**1.36**	**0.79**	**0.96**
CD4-2B	**1.01**	**1.28**	**1.00**	**1.40**	**1.13**	**1.71**	**0.77**	**1.26**
T-bet	**1.59**	**1.13**	**1.49**	**1.81**	**0.99**	**1.57**	**0.69**	**0.92**
GATA3	**1.44**	**1.31**	**1.91**	**1.56**	**1.06**	**1.31**	**0.79**	**0.91**
Foxp3a	**0.88**	**1.11**	**0.99**	**1.37**	**0.69**	**0.97**	**0.54**	**0.86**
Foxp3b	**1.00**	**1.07**	**1.13**	**1.17**	**0.85**	**1.05**	**0.71**	**0.97**
RORγ	**1.51**	**1.61**	**2.22**	**2.52**	**1.54**	**1.92**	**1.83**	**1.49**
CD28	**0.80**	**0.94**	**0.91**	**0.98**	**0.81**	**1.08**	**0.64**	**1.09**
CTLA4	**1.38**	**1.57**	**1.16**	**0.84**	**0.77**	**1.00**	**0.58**	**0.81**
CD80/86	**1.02**	**1.25**	**1.18**	**1.25**	**1.08**	**1.31**	**0.45**	**1.59**
CD209 like	**1.04**	**1.54**	**1.34**	**0.97**	**0.59**	**1.04**	**0.77**	**1.58**
LAMP3	**3.03**	**1.06**	**1.26**	**1.07**	**0.54**	**0.75**	**0.87**	**0.96**
MHCI	**0.77**	**1.15**	**1.42**	**1.80**	**1.32**	**1.26**	**0.76**	**0.96**
MHCII	**1.24**	**1.14**	**1.89**	**2.20**	**0.91**	**1.06**	**1.28**	**1.34**
sIgM H	**1.14**	**1.37**	**1.27**	**1.27**	**0.79**	**0.90**	**1.47**	**1.31**
mIgM H	**1.59**	**1.81**	**1.82**	**1.51**	**0.82**	**1.03**	**1.14**	**1.33**
sIgT H	**1.09**	**1.82**	**1.60**	**0.83**	**0.47**	**1.49**	**0.86**	**1.41**
mIgT H	**0.84**	**1.92**	**1.37**	**0.91**	**0.75**	**0.94**	**0.86**	**1.37**
sIgD H	**0.59**	**1.26**	**1.03**	**0.58**	**0.84**	**1.04**	**0.77**	**1.04**
mIgD H	**1.09**	**1.52**	**0.97**	**0.85**	**0.82**	**1.00**	**0.74**	**1.35**
Cytokines and SOCS genes								
IL-4A	**1.31**	**2.07**	**0.92**	**1.16**	**0.93**	**1.03**	**1.69**	**0.90**
IL-4B1	**0.84**	**0.99**	**0.79**	**0.70**	**0.48**	**0.90**	**0.79**	**0.87**
IL-4B2	**1.12**	**1.15**	**0.82**	**0.78**	**0.94**	**0.46**	**0.48**	**1.29**
IL-8	**0.64**	**1.71**	**3.20**	**2.20**	**1.46**	**1.28**	**0.78**	**2.14**
TNFα1	**2.14**	**2.40**	**1.87**	**1.69**	**2.47**	**1.42**	**1.25**	**1.41**
TNFα2	**1.99**	**2.23**	**4.52**	**2.02**	**2.32**	**1.90**	**1.38**	**1.60**
TGF-β1A	**0.93**	**1.65**	**2.60**	**0.98**	**1.45**	**1.74**	**1.00**	**1.41**
TGF-β1B	**0.94**	**1.82**	**1.56**	**1.81**	**1.67**	**1.62**	**0.95**	**0.87**
M17	**1.59**	**3.14**	**4.36**	**1.41**	**2.38**	**2.73**	**0.57**	**0.85**
SOCS1	**1.41**	**1.39**	**1.23**	**1.52**	**1.27**	**1.45**	**0.83**	**1.17**
SOCS2	**1.46**	**1.59**	**2.82**	**1.34**	**2.83**	**1.78**	**1.50**	**1.22**

Numbers in red indicate significant (p<0.05, paired sample T tests) up-regulation, in blue down-regulation and in black no change. The numbers highlighted in green at the same time point indicate significant differences between samples stimulated with rIL-4/13A and rIL-4/13B2.

**Table 3:** Primers used for cloning, expression analysis and recombinant protein production of salmonid IL-4/13A and IL-4/13B genes

Gene	Primer	Sequences(5′- to 3′)	Application
**TroutIL-4/13A**	**tIL4aF1**	CCTGCTAGCACCTCAACTTCTCC	3′-RACE
	**tIL4aF2**	CCCAACCAAAGATGAAGTCGGTG A	3′-RACE
	**tIL4aR1**	GTTGTAAACCCTCAGATGTCGGAC	5′-RACE
	**tIL4aR2**	CACCTGGTCTTGGCTCTTCACAAC	5′-RACE
	**tIL4aF0**	CGTGTGGTGATGGTGAAGGGAGA	Genomic DNA PCR
	**tIL4aR3**	CCTTGGTAAAGTCATTTAACGCCAC	Genomic DNA PCR
	**tIL4aF**	ACCACCACAAAGTGCAAGGAGTTCT	Real-time PCR
	**tIL4aR**	CACCTGGTCTTGGCTCTTCACAAC	Real-time PCR
	**tIL4arF**	GCAGCGCACCGGCAGCATAG	Recombinant protein
	**tIL4arR**	TGGTTTGGAGTTGATTTTTTGG	Recombinant protein
**SalmonIL-4/13B1**	**sIL4b1F1**	TCTGCATAGCGGAGAAGATTCTGTC	3′-RACE
	**sIL4b1F2**	CGAAGAGCAGTACGGGACTTCAG	3′-RACE
**TroutIL-4/13B1**	**tIL4b1F1**	TTGAACCTTCTTCACCCCGAG	PCR cloning
	**tIL4b1R1**	ATTGGACTATGCACGATCGACAG	PCR cloning
	**tIL4b1F**	GAGATTCATCTACTGCAGAGGATCATGA	Real-time PCR
	**tIL4b1R**	GCAGTTGGAAGGGTGAAGCTTATTGTA	Real-time PCR
	**tIL4b1rF**	GCGCCCACTAAGACACCTGA	Recombinant protein
	**tIL4b1rR**	AAGTGCTGGGGTCGTTTTGTG	Recombinant protein
**SalmonIL-4/13B2**	**sIL4b2F1**	TTGAACCTTCTTCACCCCGAG	3′-RACE
	**sIL4b2F2**	ACCTTCTTCACCCCGAGGG	3′-RACE
**TroutIL-4/13B2**	**tIL4b2F1**	ACCTTCTTCACCCCGAGGG	PCR cloning
	**tIL4b2R1**	TCGACAGCGTTTCCATCAGTCC	PCR cloning
	**tIL4b2F**	GAGACTCATCTATTGCGTATGATCATCG	Real-time PCR
	**tIL4b2R**	TGCAGTTGGTTGGATGAAACTTATTGTA	Real-time PCR
	**tIL4b2rF**	GCGCCCACTAAGACATCTGA	Recombinant protein
	**tIL4b2rR**	GCAAAGCTCTAGGGTTGTTTGTG	Recombinant protein

Cytokine receptors: Both rIL-4/13A and B2 were able to modulate the expression of the receptors for IL-4/13, the IL-6 cytokine family and IFNγ (Figure 12A-12J). Eight potential receptor subunits of IL-4/13, two each of IL-4Rα, γC, IL-13Rα1 and IL-13Rα2, have been identified in salmonids [29]. Both rIL-4/13A and B2 rapidly up-regulated the expression of both IL-4Rα paralogues, with a peak of induction at 4 h by rIL-4/13B2 (10.3-fold for IL-4Rα1 and 3.9-fold for IL-4Rα2) and at 24 h by rIL-4/13A (9.0-fold for IL-4Rα1 and 5.1-fold for IL-4Rα2, Figure 12A-12B). They also upregulated the expression of IL-13Rα2 paralogues at later time points (24-96 h) with rIL-4/13A having a more prominent effect, with a 82.8-fold increase of IL-13Rα2a and 42.8-fold increase of IL-13Rα2b seen at 96 h (Figure 12E-12F). They had no effects on IL-13Rα1a expression, but down-regulated IL-13Rα1b expression at all time points (4-96 h, Figure 12C-12D). No change of γC expression was seen in the screening.

Both rIL-4/13A and B2 also rapidly up-regulated the expression of IFNγR1 [61], with a peak at 4 h (4.7-fold for rIL-4/13A and 5.6-fold for IL-4/13B2), but were less effective for IFNγR2 expression (Figure 12G-12H). The expression of GP130 [62], a signal receptor chain for the IL-6 family cytokines, was up-regulated to a moderate degree from 4-96 h by both cytokines. In contrast, the expression of IL-6Rα, the private chain of IL-6, was down-regulated at 48 h by rIL-4/13A (Figure 12I-12J). Curiously, they up-regulated at early time points (4-48 h) the expression of MCSFR2, that is highly expressed in HK primary macrophages but down-regulated the expression of the more lowly expressed MCSFR1 paralogue (Figure 12K-12L).

Cellular marker genes: HK cells are comprised of immune cell types, including T cells, B cells, macrophages/dendritic cells. The expression of marker genes of these cell populations was examined during the course of rIL-4/13 stimulations. CD4-1 expression was increased at 24-96 h by rIL-4/13A (up to 3.7-fold) and at 48 h by IL-4/13B2 (2.2-fold) (Figure 12M). A small increase of the expression of CD8α and CD8β was observed at 4 h by rIL-4/13A and rIL-4/13B2, respectively (Figure 12N-12O). The expression of the master transcription factors for Th cell development, T-bet, GATA3 [63], FoxP3 [64] and RORγ [65], and T cell co-receptors, CD28 and CTLA, were not modulated relative to time-matched controls or were changed marginally at some time points (Table 2). The expression of B cell markers, the membrane form of IgM, IgT and IgD, and the secreted form of IgM and IgD was refractory to rIL-4/13 proteins. However, a small increase (less than 2-fold) in secreted IgT transcript expression was observed at 4 h and 48 h by IL-4/13B2 (Table 2).

Several genes relevant to the function of antigen-presenting cells (macrophages/dendritic cells) were induced. CD83 [66] expression was rapidly induced at 4-48 h by rIL-4/13B2 with a peak at 4 h with a 9.3-fold increase, and by rIL-4/13A at 24 h (Figure 12P). The expression of CLEC4, a trout DC-SIGN/CD209 like molecule [67] was also rapidly induced at 4-96 h and peaked at 4 h (4.8-fold) with rIL-4/13A stimulation, and to a lesser extent by rIL-4/13B2 at 4 h and 96 h (Figure 12Q). Another CD209 like molecule (CD209L2) was also found to be up-regulated by both rIL-4/13 proteins at the later time points (48-96 h) (Figure 12R). The expression of CD209L1, and the MHC class II beta chain was refractory to IL-4/13 stimulation. However, small changes in expression of other genes, ie LAMP3 [68], CD80/86 [69] and MHC class I, were seen at the late time points (Table 2).

### Cytokines and suppressor of cytokine signalling (SOCS) genes

Induced changes in cytokine gene expression of the paralogues of IL-4/13, TGF-β1, TNFα and IL-8 were minor (Table 2), but for IFNγ, IL-1β and IL-10 they were relatively large and will be described in more detail. Two IFN-γ paralogues are present in salmonids. IFNγ1 expression was down-regulated at 24-96 by rIL-4/13A and at 96 h by rIL-4/13B2. Similarly, IFNγ2 expression was down-regulated at 48-96 h by rIL-4/13A and at 4 h and 96 h by rIL-4/13B2 (Figure 13A-13B).

Three IL-1β paralogues are present in salmonids with IL-1β1 highly expressed in HK cells and HK primary macrophages [41]. IL-1β1 expression was also significantly down-regulated at 48-96 h by rIL-4/13A and at 24 h and 96 h by rIL-4/13B2 (Figure 13C).

LECT2 (Leukocyte cell-derived chemotaxin-2) acts as a chemotactic factor for neutrophils and promotes inflammation and activation of macrophages [70-71]. LECT2 expression was significantly down-regulated from 24 h to 96 h by both rIL-4/13 proteins (Figure 13D)

Two paralogues of the anti-inflammatory cytokine IL-10 are present in salmonids [72]. Low level induction of IL-10a expression was observed at 48 h by rIL-4/13A and at 96 by rIL-4/13B2 (Figure 13E). In contrast, a prominent induction of IL-10b expression was observed from 4-96 h by both cytokines, that peaked at 24 h (7.1-fold) for rIL-4/13B2 stimulation and at 96 h (25.2-fold) for rIL-4/13A stimulation (Figure 13G). The rIL-4/13 proteins also induced the expression of another anti-inflammatory cytokine TGF-β1 but with less potency (Table 2).

SOCS are cytokine-inducible negative regulators of cytokine signaling. SOCS3 [73] expression was highly induced from 4-96 h and peaked at 24 h with a 30.3-fold increase for rIL-4/13A stimulation and a 18.5-fold increase for rIL-4/13B2 stimulation (Figure 13F). A small increase of SOCS2 expression was also seen but no change in SOCS1 expression occurred with either protein (Table 2).

### Rainbow trout IL-4/13 isoforms increase IgM secreting B cells but have no effects on IgM^+^ B cell proliferation *in vitro*

**Figure 14:**
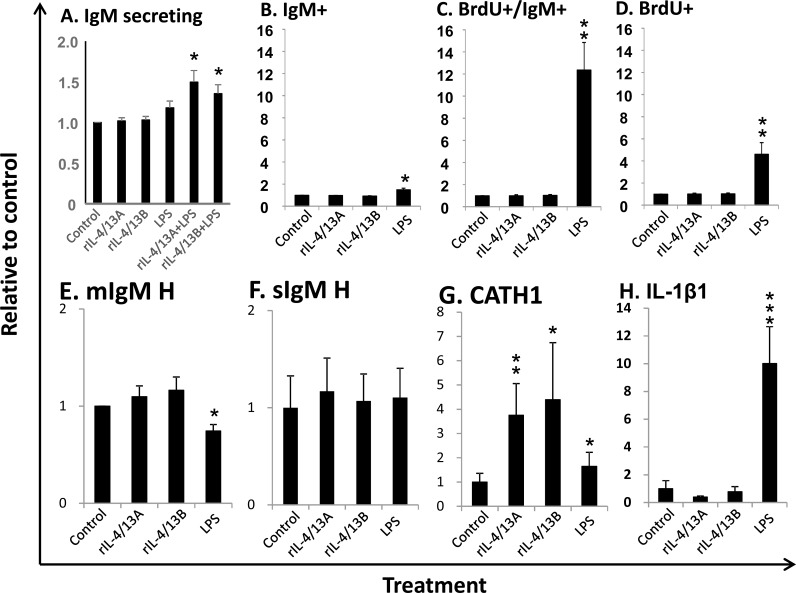
Effects of rIL-4/13 isoforms on IgM+ B cells A. Spleen leucocytes were incubated with/without rIL-4/13A (200 ng/ml), rIL-4/13B2 (200 ng/ml), LPS (100 μg/ml), or LPS in combination of rIL-4/13A or IL-4/13B2 for 3 days at 20°C and IgM secreting cells were determined by ELISPOT. The IgM secreting B cells (means + SEM, *n* = 5) relative to control (= 1) are shown. B-D. Spleen leucocytes were incubated with/without rIL-4/13A, rIL-4/13B2, or LPS as positive control for 3 days at 20°C. The proliferating cells were then labeled with BrdU and incubated for a further 24 h. The proliferating (BrdU^+^) cells and IgM^+^ B cells were then determined by flow cytometry with typical results shown in Figure S12. The relative number (means + SEM, *n* = 11) of IgM^+^ B cells (**B**), proliferating B cells (BrdU and IgM double positive, C) and total proliferating cells (BrdU^+^, D) are shown. E-H. Spleen leucocytes (*n* = 6) were incubated with/without rIL-4/13A, rIL-4/13B, or LPS as positive control for 24 h at 20°C. IgM^+^ cells were sorted into TRIzol. The relative transcript expression (means + SEM, *n* = 6) of the membrane form of IgM heavy chain (**E**), secretory form of IgM heavy chain (**F**), CATH1 (**G**) and IL-1β (**H**) in FACS sorted IgM^+^ B cells are shown. The relative significance of paired sample T tests between stimulated and control samples are shown above the bars as: *p **≤** 0.05 **p **≤** 0.01 and ***p **≤** 0.001.

Previous analysis revealed that trout B cells isolated from spleen expressed higher levels of potential receptor subunits of IL-4/13 isoforms, e.g. IL-4Rα2, γC1 and IL-13Rα2a [29], compared to B cells isolated from blood and head kidney. There are more IgM^+^ B cells in the lymphocyte population in spleen (30.3%) than in the HK (12.4%) [74]. Thus, B cells from spleen were used throughout this study. A mean of 160 IgM secreting cells could be detected in each well of control cells (*n* = 5) by ELISPOT. No significant effects were observed when the cytokines and LPS were added alone. However, the combination of LPS with rIL-4/13A or rIL-4/13B2 significantly increased the number of IgM secreting B cells (Figure 14A). We further analysed the potential of these cytokines to induce IgM^+^ B cell proliferation (*n* = 11). In the control cells, the average IgM^+^, BrdU^+^/IgM^+^ and BrdU^+^ cells were 29.44%, 0.35% and 1.02%, respectively (Figure S12). Unexpectedly, neither IgM^+^ cells nor proliferating IgM^+^ cells (BrdU positive) were increased after incubation with rIL-4/13 isoforms, despite a significant increase of both (1.5 fold for IgM^+^ cells and 12.4 for proliferating IgM^+^ cells) by LPS (Figure 14B, 14C). Furthermore, IL-4/13 isoforms had no effects on total BrdU positive cell numbers in spleen leucocyte cultures whilst LPS stimulation increased these cells by 4.6-fold relative to the control leucocytes (Figure 14D).

To confirm the effectiveness of these treatments, the expression of several marker genes was analysed in FACS-sorted B cells isolated from spleen leucocytes incubated with/without rIL-4/13A, rIL-4/13B2 and LPS for 24 h. Trout IL-4/13 isoforms significantly increased the expression of CATH1, but had no effects on transcript expression of IgM H-chains (membrane- and secretory forms), and IL-1β1 (Figure 14E-14H). As expected, LPS up-regulated the expression of IL-1β1 and CATH1 [42], down-regulated the expression of the membrane form of the IgM H-chain, but had no effect on the secretory IgM H-chain transcript. Taken together, these results show that the trout IL-4/13 isoforms increase IgM secreting B cell number but have no effects on proliferation of IgM^+^ B cells *in vitro*.

## DISCUSSION

In this study, four genomic loci have been identified in salmonids and three active IL-4/13 paralogues belonging to the two types of fish specific type-2 IL-4/13 genes have been cloned in both rainbow trout and Atlantic salmon. Our molecular characterisation of these genes suggests lineage-specific evolution of type 2 cytokines in vertebrates. We next extensively compared the expression and modulation of all the IL-4/13 paralogues in a single fish species *in vivo* and *in vitro*, providing a first comprehensive insight into their functional roles in fish. We then compared the function of the two types of fish-specific type-2 cytokines in fish for the first time, and this revealed shared but also distinct bioactivities. This study will facilitate a broader understanding of the evolution of type-2 immunity in vertebrates, the functional characterisation of fish-specific type-2 cytokines and the future potential to manipulate type-2-like immunity to protect fish from infectious disease.

### Lineage-specific evolution of type-2 cytokines

A single IL-4/IL-13 gene was likely to have existed in ancestral Gnathostomes, which has been duplicated in different lineages by WGD and/or tandem gene duplication events [10]. Thus, tandem gene duplication in mammals and birds led to the evolution of IL-4 and IL-13, and WGD in 3R teleost fish led to the IL-4/13A and IL-13B paralogues [10, 32]. A further 4R WGD in salmonids potentially produced four IL-4/13 paralogues. However, due to pseudogenisation of one of the two IL-4/13A loci, only three active paralogues, IL-4/13A, B1 and B2 are present in Atlantic salmon and rainbow trout (Figures 1, S1-S7).

The IL-4, IL-13 and IL-4/13 genes are fast evolving. This is demonstrated by the low homology within each lineage and between molecules. Mammalian IL-4 and IL-13 only share low aa identities, of 40-57% and 53-64% between humans, cows and mice, respectively. These are remarkably lower compared to mammalian IL-7 (61-75%), IL-15 (72-78%) and IL-21 (59-68%), (Table S1). Furthermore, the homology between IL-4, IL-13 and IL-4/13 is low. For example, IL-4 and IL-13 only share 20-23% aa identity in mammals, and 20-27% in birds. The cartilaginous and teleost fish IL-4/13 molecules share only 16-29% aa identity to mammalian IL-4 and 15-28% identity to mammalian IL-13 (Table S1). Such rapid evolution is also exemplified by salmonid IL-4/13 molecules. In a similar manner to salmonid IL-4/13, three paralogues of IL-1β and IL-12 p35 have also been cloned in both salmon and trout, which share the same origin. The aa identities between 4R orthologues (eg A, B1, B2), 4R paralogues (eg B1 and B2), and 3R paralogues (eg. A and B1 or B2) are 90-95%, 75-79% and 29-32% for IL-1β molecules [41]; 95-97%, 87-90% and 36-39% for IL-12 p35 [43]; but only 82%, 62-66% and 26-29% for IL-4/13 molecules (Table 2), respectively.

The orthologues are typically grouped together and separate from the other paralogues from a WGD in phylogenetic tree analysis, as seen with the two types of IL-1β [41], TNFα [42], IL-12 [43] and CISH [75] that arose from the 3R WGD. However, in the case of IL-4/13A and IL-4/13B the orthologues do not group together, despite a well-supported 4R WGD orthologues-grouping of these molecules in salmonids (Figure S9). A lineage-specific grouping is apparent, eg bird or mammalian IL-4 and IL-13, and IL-4/13 molecules from different teleost fish lineages. The gene organisation, in terms of the size of exon 3 (Figure 2), and disulfide bonding potential of the peptides (Figures 3 and S10) are also lineage-specific. Such data provide support for a lineage-specific evolution of the type-2 cytokines in different vertebrates groups.

### Differential expression of the type 2 cytokines IL-4/13A and IL-4/13B

Differential expression and modulation of the paralogues may suggest a sub-functionalization and/or neofunctionalisation [75]. Our extensive analysis of gene expression revealed that different isoforms of the IL-4/13 paralogues are differentially expressed and modulated. Trout IL-4/13A is the major isoform highly expressed constitutively in tissues. Its expression is also detectable in all the four cell lines examined, in purified B cells, HK cells and primary macrophages (Figure 4). However its expression is less responsive to stimulation and infection (Figures. 5, 6, 8, 9). These patterns of expression and modulation may suggest a homeostatic role of this IL-4/13 isoform to provide a basal or house-keeping level of type-2 (IL-4/13) function.

Although the constitutive expression level of IL-4/13B1 and B2 isoforms are lower compared to that of IL-4/13A, they are the major isoforms that are highly induced by viral and parasitic infection *in vivo* and by PAMPs in primary macrophages. They are also the major isoforms that respond to the T cell mitogen PHA and cytokines with major effects on T cells (Figure 8). Furthermore, IL-4/13B1 and B2 expression is not detectable in cell lines of non-immune cells, or in B cells, but is present in the macrophage cell line RTS-11, in primary macrophages and in HK cells (Figure 4). These data suggest that trout IL-4/13B1 and B2 isoforms are the major inducible isoforms that are expressed in T cells and other immune cells, and likely have a major role in T cell immunity in salmonids.

In agreement with the induced expression *in vivo* by viral and parasitic infection, the expression of trout IL-4/13B paralogues is induced in antigen presenting cells (ie the primary HK macrophages) by PAMPs (Figure 9). In addition, HK cells were found to highly induce the expression of IL-4/13B paralogues in response to formalin killed bacteria, whether pathogenic or non-pathogenic, and Gram positive or Gram negative (Figure 8). These data show that infected rainbow trout are able to up-regulate IL-4/13 expression in response to PAMPs from viruses, parasites and bacteria. The *in vivo* down-regulation of IL-4/13 paralogues in non-vaccinated fish during *Y. ruckeri* infection (Figure 7) could be indicative of mechanisms by which fish pathogens evade immunity.

Interestingly, the vaccinated fish expressed higher levels of the IL-4/13 paralogues than naïve fish during early stages of the disease, at 24 h and 48 h post *Y. ruckeri* infection in gills and HK (Figure 7). Whether this would also occur in other major secondary lymphoid organs such as the spleen remains to be determined. The functional role of IL-4/13 paralogues in this model of vaccine mediated protection is difficult to assess without IL-4/13 knockout trout lines. However, perhaps if the infection is under control, the pathogen is unable to evade immunity in vaccinated fish with the expression of IL-4/13 paralogues being maintained. Thus, the expression of IL-4/13 paralogues could be utilized as a novel molecular marker in evaluating vaccine efficacy. This may be especially important when designing a multi-valent vaccine or vaccines to enable cross protection.

### The type-2 cytokines IL-4/13A and IL-4/13B have overlapping but also distinct bioactivity

By sharing receptors, mammalian IL-4 and IL-13 have overlapping but also distinct functions [1-2]. The orthologues of all mammalian receptor subunits are present in fish with two copies of each present in salmonids, which are differentially expressed and modulated in different tissues and cell lines [27-29]. Although the specific pairing of the ligands (three in salmonids) and receptors (eight in salmonids) is not clear in fish, the outcome of cytokine stimulation in a cell should be determined by 1) the presence of a combination of different receptor subunits on cell surfaces, 2) the affinity of the ligand/receptor binding, which will determine the strength of the signalling, 3) the presence of other cells that may compete for ligand binding (but giving a different outcome), and 4) the microenvironment that may contain soluble receptors or other molecules that can regulate ligand/receptor binding or signalling.

Both rIL-4/13A and IL-4/13B2 modulated the expression of cellular markers of T cells and macrophages/dendritic cells indicating these are target cells that express relevant receptors. This is similar to mammalian IL-4 that acts on a variety of cell types [76]. The distinct bioactivities between rIL-4/13A and rIL-4/13B2 observed in HK cells are mainly the result of the magnitude of the response or differential kinetics (Figures 10, 11, 12, 13). These differences are likely explained, at least in part, by differences in receptor usage or affinity to different receptor complexes expressed on different cell populations. The dissection of the mechanisms will require the development of new molecular tools to isolate different cell populations from HK cells in the future.

AMPs and APPs are evolutionarily conserved effector molecules of the innate immune system that have important roles in the resolution of infection and activation of the adaptive immune response. The rapid up-regulation of the expression of SAP1, hepcidin and CATH1 by both IL-4/13A and IL-4/13B2 suggests a role of these type-2 cytokines in the resolution of infection. Rapid up-regulation of trout IL-4Rα1 and IL-4Rα2 expression by rIL-4/13A and B2 (Figure 12) was also seen and may suggest a self-amplification mechanism of signalling specific to these cytokines. Interestingly, the expression of the potential decoy receptors IL-13Rα2a and α2b was significantly up-regulated at late stages (24-96 h) of stimulation of HK cells, with rIL-4/13A having more prominent effects compared with rIL-4/13B2 (Figure 12).

Treatment of HK cells with rIL-4/13A and B2 resulted in the down-regulation of the proinflammatory cytokines IFN-γ1, IFN-γ2 and IL-1β1, at the late time points, but up-regulation of anti-inflammatory genes including IL-10a, IL-10b, SOCS3 (Figure 13), and (to a lesser extent) SOCS2 (Table 2), perhaps as a negative feedback to limit the signalling of fish type-2 cytokines. Clearly these results suggest an anti-inflammatory role of fish type-2 cytokines, as seen with mammalian IL-4 and IL-13 that are known to suppress the expression of proinflammatory genes [76]. There is also the potential for inhibition of Th1 type responses in fish by IL-4/13, as seen with IL-4 in mammals. Interestingly, the expression of several cytokine receptors was also affected. For example, IL-6R, and MCSFR1 were down-regulated, especially by rIL-4/13A (Figure 12). IL-6 signals *via* a complex of the common chain gp130 and the private IL-6Rα, and is involved in inflammation and macrophage activation and proliferation in trout [62]. MCSFR1 and MCSFR2 are potentially the receptors for at least three fish cytokines, MCSF1, MCSF2 and IL-34, which regulate the mononuclear phagocyte system with crucial roles in inflammation, and in maintaining organismal homeostasis [77-78]. In contrast, IFNγR1, and to a lesser extent IFNγR2, MCSFR2 and gp130 were also up-regulated to some extent. Modulation of the expression of these receptors by rIL-4/13A and B2 indicates an important role in the fish cytokine network *via* cross talk with the IFN-γ and IL-6 family members.

### The functional role of IL-4/13 isoforms in B cells

Mammalian B cells first produce IgM and IgD and then, after activation, they can switch to produce IgG, IgA, or IgE. IL-4 can regulate Ig class switching in mice to IgG1 and IgE, and in humans to some subclasses of IgG and to IgE [79]. Teleost fish also produce IgM and IgD but also a unique Ig termed IgT, with IgM and IgD co-expressed on the same cells but IgT on separate B cells [80]. Thus class-switching per se may not be a function of Th2 type cytokines in bony fish. The trout rIL-4/13A and B2 had no effect on transcript expression of the secreted or membrane forms of IgM and IgD in HK cells (Table 2), or on the proliferation of spleen leucocytes and IgM^+^ B cells (Figure 14) *in vitro*. However, both cytokines increased the numbers of IgM secreting B cells, as seen in ELISPOT assays. Thus trout type-2 cytokines on their own have no direct proliferative effects for spleen leucocytes *in vitro*, including IgM^+^ B cells, but seem to be a survival or reactivation factor for IgM secreting B cells. Interestingly, in mammals, these “type-2 immune response-related” cytokines are not only produced by CD4 Th2 cells but also by ILC2s [12] that play an important role in early type-2 responses. In mice, ILC2 can be activated in response to IL-4 produced by basophils releasing large amounts of Th2 cytokines such as IL-5 and IL-13, as well as IL-6 [81]. This cytokine production together with the interaction of ICOS (expressed on ILC2 cells) and ICOSL (expressed on the surface on B lymphocytes) has been shown recently to increase the survival and promote the production of IgE by B cells [82-83]. Furthermore, IL-13 produced by ILC2 has been shown to activate non-dividing plasma cells [84], and ILC-2-derived IL-5 and IL-6 have been shown to support self-renewal of B1 cells from the peritoneal cavity and Ig production by splenic B cells [85]. This does not rule out the possibility that the trout type-2 cytokines could have proliferative effects in the presence of additional factors that are not present in the culture medium *in vitro.* Interestingly, a recent report in zebrafish demonstrated that i.p. administration of rIL-4/13A significantly increased the number of B cells in PBL [39]. However, whether the increased number of B cells is the result of IL-4/13 proliferation effects (*in vivo*), or indirect effects (eg *via* IL-4/13 induced factors) is not known and will be interesting to study further. The effects of type-2 cytokines on IgT^+^ B cells in fish also needs future investigation.

### Implications towards the evolution of type-2 immunity in early vertebrates

Th2-type immunity is hypothesised to give a fitness advantage by reduction of collateral tissue damage from metazoan parasites, and acts as a counter-regulator to Th1 responses that are primarily antimicrobial [26]. Whilst Th2 responses have been known for a long time [2], there are still many questions relating to the mechanisms that initiate and control these responses in mammals [86], which comparative studies in other vertebrates may aid. There are two types of type-2 cytokines present in teleost fish, IL-4/13A and IL-4/13B [10], that may be further increased in some species by gene duplication or WGD (eg in salmonids, as demonstrated in this study). Fish type-2 cytokines have overlapping bioactivities, such as the induction of APPs, AMPs and IL-10, and inhibition of proinflammatory cytokines (IFN-γ and IL-1β), that could be due to conserved receptor usage [28-29] as seen in mammals with IL-4 and IL-13. Fish IL-4/13A is broadly and highly expressed and has potent bioactivities at low concentrations, which may provide a basal level of type-2 immunity in fish. Fish IL-4/13B exhibits low constitutive levels but is highly inducible in response to PAMPs and T cell stimulants (mitogens and cytokines) with its expression perhaps being more restricted to immune cells. Its maximal bioactivity required relatively high concentration that may reflect low levels of its preferred receptor in unstimulated HK cells. In carp, a Th2-like cell clone that expresses a high level of IL-4/13B but not IL-4/13A after PHA stimulation has been identified recently, supporting the concept that IL-4/13A and IL-4/13B can be secreted by different cell types [37]. Mammalian IL-4/IL-13 expression is controlled by chromatin remodelling in the locus control region within the 3′ end of the RAD50 gene, and a potential regulatory element within the KIF3A gene [12]. The linkage to RAD50 (IL-4/13A) or KIF3A (IL-4/13B) likely reflects the need for regulatory elements associated within these genes in fish, but the loss of the linkage to both has perhaps impacted on the unique expression patterns seen in trout outlined above. Heterogeneity of Th2 cell responses are known in mammals, as seen with the so-called “inflammatory Th2 cells” and “noninflammatory Th2 cells” [83], with interest in the potential to re-programme subsets that may cause pathology to a “protective” phenotype. Understanding the origins of these responses and the different Th2 phenotypes present in other vertebrate groups may be informative in this regard. Overall, these data suggest an enhanced type-2 immunity in fish can occur following detection of particular danger signals and results in the activation and expression of the IL-4/13B gene(s), which may be more important for specific T cell mediated immunity, at least in salmonid fish.

## MATERIALS AND METHODS

### Fish

Rainbow trout were maintained in aerated fibreglass tanks supplied with a continuous flow of recirculating freshwater at 14°C. Fish were fed twice daily on a commercial pellet diet (EWOS), and were given at least 2 weeks of acclimatization prior to treatment. All the experiments described comply with the Guidelines of the European Union Council (2010/63/EU) for the use of laboratory animals, and were carried out under UK Home Office project licence PPL 60/4013, approved by the ethics committee at the University of Aberdeen.

### Cloning of the IL-4/13 genes in salmonids

#### Cloning of salmonid IL-4/13A cDNA and DNA

Four salmon expressed sequence tag (EST) entries (acc. nos. EG837624-25 and EG860817-18) were identified that encode for salmon IL-4/13A. To obtain trout IL-4/13A cDNA, primers (tIL4aF1 and 2, Table 3) were designed according to the salmon ESTs and used for 3′-RACE (Rapid amplification of cDNA ends) using trout gill SMART cDNA, as described previously [87]. A 0.75 kb 3′-RACE product was obtained and contained the trout IL-4/13A CDS and 3′-UTR. A 0.45 kb 5′-RACE product was obtained using primers (tIL4aR1 and R2, Table 3) designed on the trout IL-4/13A sequence. The overlapping cDNA sequence (acc. no. FN820500) contained the 5′-UTR, complete CDS and the 3′UTR. The PCR protocol, cloning of the PCR product and sequence analysis was as described previously [57, 78]. Fish IL-4/13A gene is closely linked to RAD50 [19, 32]. A 4.39 Kb genomic DNA (acc. no. FN820501) was amplified using primers located on RAD50 and in the 3′-UTR of trout IL-4/13 cDNA.

#### Cloning of salmonid IL-4/13B1 and IL-4/13B2 cDNA

The Atlantic salmon whole genome shotgun (WGS) sequence was searched (TBLASTN [88]) using known fish IL-4/13 molecules. Candidate WGS contigs (Table 1) were identified and exons predicted as described previously [43]. Primers (Table 3) were designed against the predicted 5′-UTR and used for 3′-RACE using SMART cDNA as above. The cloning of the PCR products yielded full-length cDNA sequences from two salmon IL-4/13 genes designated as IL-4/13B1 and IL-4/13B2, according to their linkage to the KIF3A gene [10, 32]. The trout counterparts of the salmon IL-4/13B1 and B2 were amplified from a mixed tissue cDNA sample using primers designed in the 5′- and 3′-UTR of the salmon sequences (Table 3), and then were cloned and sequenced. The genomic sequences of trout IL-4/13B genes were identified from the recent release of the trout WGS contigs [89].

### Sequence analysis

The nucleotide sequences generated were assembled and analyzed with the AlignIR programme (LI-COR, Inc.). Homology search was performed at the National Center for Biotechnology Information (NCBI) using the BLAST program (http://blast.ncbi.nlm.nih.gov/Blast.cgi) [85]. The gene organization was predicted using the Spidey program at NCBI. Protein prediction was performed using software at the ExPASy Molecular Biology Server (http://www.expasy.org/tools) [60] and signal peptides were predicted using the SignalP4.0 program [90]. Disulfide bonding and cysteine connectivity were predicted using the DISULFIND program (http://disulfind.dsi.unifi.it) [49]. Global sequence comparison was performed using the scoring matrix BLOSUM62 within the MatGAT program, with a gap open penalty of 10 and gap extension penalty of 1. [91]. Multiple sequence alignments were generated using CLUSTALW [92]. The synteny of IL-4/13 loci was analysed using Genomicus (database version 75.01) [93]. A neighbour-joining phylogenetic tree was constructed on full-length amino acid multiple alignments using the MEGA6.0 software [44]. The evolutionary distances were computed using the JTT matrix-based method with all ambiguous positions removed for each sequence pair.

### Transcript distribution of rainbow trout IL-4/13 paralogues in tissues, cell lines and purified immune cells

RNA preparation, cDNA synthesis, and real-time PCR analysis were performed as described previously [29, 57]. The primers (Table S2) for real-time-PCR were designed so that at least one primer crossed an intron, to ensure that genomic DNA could not be amplified under the PCR conditions used. To directly compare the expression level of the different IL-4/13 paralogues, a reference was constructed using equal molar amounts of PCR product from each gene, including the house keeping gene elongation factor-1α (EF-1α). The relative expression level of each sample was normalized against the expression level of EF-1α.

#### Tissues from healthy fish

Six healthy rainbow trout (∼140 g) were killed and seventeen tissues (blood, thymus, gills, scales, skin, muscle, tail fins, adipose fin, brain, adipose tissue, spleen, liver, heart, intestine, gonad, head kidney and caudal kidney) were collected and processed as described previously [41, 94]. The RNA preparation, cDNA synthesis and real-time PCR analysis of gene expression was also as described previously [29, 61].

#### Cell lines, head kidney (HK) cells, primary macrophages and purified IgM^+^ B cells

Four rainbow trout cell lines, a monocyte/macrophage-like cell line RTS-11 from spleen [95], an epithelial cell line RTL from liver [96], and fibroid cell lines RTG-2 from gonad [97] and RTGill from gills [98], were cultured in Leibovitz medium (L-15, Life Technologies) supplemented with 100 IU/ml penicillin/100 μg/ml streptomycin (P/S), and 10% fetal calf serum (FCS). The cells were dissolved in TRI reagent (Sigma) for total RNA preparation, with real-time RT-PCR quantification of gene expression performed as above. Preparation of HK cells, primary macrophages and B cells will be described in the next section.

### Preparation of HK cells, primary HK macrophages, spleen leucocytes and IgM^+^ B cells

Heparinized blood was extracted from freshly killed rainbow trout and HK and spleen tissue then collected and placed into L-15 medium supplemented with P/S, 10 units/ml heparin and 5% FCS. Single cell suspensions were generated using 100 μm nylon cell strainers (BD Biosciences). The HK cells were then washed and used directly for *in vitro* stimulation [57] or for preparation of primary macrophages as described previously [59]. To prepare spleen leucocytes, the splenocytes were placed onto 30%/51% Percoll (GE Healthcare) discontinuous density gradients and then centrifuged at 500 g for 30 min at 4°C. The interface cells were collected and washed twice with L-15 containing 5% FCS. The IgM^+^ B cells were purified by FACS sorting of spleen leucocytes using an anti-trout IgM mAb (1.14) [74].

### Expression of trout IL-4/13 paralogues during viral infection

VHSV has been isolated from more than 60 fish species, with rainbow trout the most susceptible [50]. The kidney is one of the major targets of VHSV infection, and thus the expression was examined in the kidney. The pathogenic VHSV strain DK-F1 was used for infection and the challenge performed as described previously [57, 99]. Fish were i.p. injected with 1 × 10^8^ TCID50/fish or the equivalent volume of medium alone as a control. Four fish from each group were killed at 1, 2, 3, 4, 5, 7, 9 and 12 days post-infection and HK tissues collected. Real-time PCR analysis was conducted as described above.

### Expression of trout IL-4/13 paralogues during parasitic infection

*T. bryosalmonae* infects salmonid fish primarily *via* the gill epithelia, subsequently gaining access to internal tissues *via* the vascular system, with the kidney being the main target organ [51]. Trunk kidney tissue collection and cDNA preparation was as described previously [52]. The severity of clinical pathology was analysed and a kidney swelling grade assigned to each fish according to the kidney swelling index system devised by Clifton-Hadley and colleagues [51]. Real-time PCR analysis was conducted as described above.

### Modulation of the expression of trout IL-4/13 paralogues by vaccination and bacterial challenge

Archived material of a vaccination and challenge experiment against ERM [53] was used. Briefly, a group of 50 trout were vaccinated with AquaVac^TM^ ERM by i.p. injection of 0.1 ml of vaccine as recommended by the manufacturer (Intervet Schering-Plough). An additional group of 50 trout that were handled in the same way but without vaccination served as controls. The fish were left for 60 days and then challenged by i.p. injection of a pathogenic strain (MT3072) of *Y. ruckeri* (0.5 ml/fish, 1×10^6^ cfu/ml) or 0.5 ml of PBS as control. This dose of MT3072 has been shown to kill ∼90% of fish in previous independent pre-experimental tests, with mortalities occurring from day 3. In this trial four groups of 25 fish were established; the control fish injected with PBS (Naïve/PBS), or *Y. ruckeri* (Naïve/YR), and vaccinated fish injected with PBS (Vac/PBS) or *Y. ruckeri* (Vac/YR). Five fish from each group were killed at 6 h, 24 h, 48 h and 72 h after challenge/PBS injection, to assess the responses prior to the expected onset of mortalities in the Naïve/YR group. The head kidney and gills were collected for gene expression analysis as above.

### Modulation of the expression of trout IL-4/13 paralogues in HK cells and primary HK macrophages

Freshly prepared HK cells (isolated as described above) were stimulated with formalin killed *A. davidanieli* (100 μg/ml, wet weight), *R. salmoninarum* (100 μg/ml), *A. salmonicida* (100 μg/ml) and *Y. ruckeri* (100 μg/ml); a T cell mitogen (PHA at 5 μg/ml), and two recombinant cytokines (rIL-2 at 200 ng/ml [56] and rIL-21 at 100 ng/ml) [57] for 4 h, 8 h, 24 h, 48 h and 96 h. Four day old primary HK macrophages [59] were stimulated with PAMPs (poly I:C at 50 μg/ml and peptidoglycan (PGN) at 10 μg/ml), and recombinant trout cytokines (rIL-1β at 20 ng/ml [58], rIL-6 at 200 ng/ml [59], rIFN-γ at 20 ng/ml [29], and TNFα at 10 ng/ml [42]) for 4 h, 8 h and 24 h. The concentration chosen for each stimulant was deemed optimal for immune gene expression studies based on our previous work [29, 42, 56-59]. The stimulation was terminated by dissolving the cells in TRI reagent (Sigma, UK). Quantification of gene expression was as described above. Modulated expression was expressed as a fold change calculated as the mean expression levels in stimulated cells normalized to that of time-matched controls.

### Cloning, expression and purification of recombinant trout IL-4/13 isoforms

#### Cloning

The sequences encoding the mature peptides of trout IL-4/13 isoforms were amplified from cloned cDNA using the primers detailed in Table 3. The amplified products were cloned to a pET vector (Novagen) as described previously [57, 59]. Each construct has an ATG codon added at the N-terminus for translation initiation and a sequence encoding for GSGHHHHHHHH added at the C-terminus for purification. Thus, the recombinant trout IL-4/13A, B1 and B2 were 133 aa, 145 aa and 148 aa, with a calculated molecular weight/theoretical pI of 15.15 kDa/7.25, 16.51 kDa/7.82 and 16.76 kDa/7.75, respectively.

#### Expression and purification of trout IL-4/13 isoforms

For each protein a sequence confirmed plasmid was transformed into BL21 Star (DE3) competent cells (Invitrogen). The induction of recombinant protein production, purification under denaturing conditions, refolding, re-purification under native conditions, SDS-PAGE analysis of proteins and quantification of protein concentration were as described previously [57, 59, 100]. The refolding buffer contained 25 mM MES (MW), pH6.5 (6.5), 25% glycerol, 0.6 M arginine monohydrochloride, 0.5% Triton-100, 0.2% PEG3350, 10 mM 2-mercaptoethanol and 1 mM EDTA. The purified proteins were desalted in desalting buffer (DSB) (50 mM Hepes, pH7.0, 140 mM NaCl, 10 mM arginine, 50% glycerol and 5 mM 2-ME) using PD-10 Desalting Columns (GE Healthcare). After sterilization with a 0.2 μm filter, the recombinant proteins were aliquoted and stored at −80°C ready for stimulation of cells.

### Modulation of gene expression in HK cells by recombinant trout IL-4/13 isoforms

The recombinant proteins produced above were initially added to HK cells and splenocytes (2×10^6^ cells/ml) at 200 ng/ml for different times (4 h to 24 h) to test their bioactivity in terms of modulating gene expression as described previously [42, 101]. Further tests were conducted using HK cells with different doses (0.01 ng to 1,000 ng/ml) for 4 h, or 200 ng/ml of recombinant proteins for different times (2 h to 96 h). The experiments were terminated by dissolving the cells in TRI reagent and real-time PCR analysis was conducted as described above. After an initial screening of over 200 genes, the expression of 62 trout immune genes, including those encoding for cellular markers, cytokines and cytokine receptors, were analysed in the time course experiment using primers detailed in Table S2.

### The Enzyme-Linked ImmunoSpot (ELISPOT) assay

ELISPOT was used to quantify the number of IgM-secreting B cells. ELISPOT plates containing Inmobilon-P membranes (Millipore) were activated with 70% ethanol for 30 s, coated with anti-trout IgM mAb (clone 4C10) at 2 μg/ml, diluted in PBS and incubated overnight at 4°C. To block non-specific binding to the membrane, plates were then incubated with PBS containing 2% bovine serum albumin (BSA) for 2 h at room temperature. Stimulated (with rIL-4/13A, rIL-4/13B2, LPS, or LPS in combination with rIL-4/13A or rIL-4/13B2) or unstimulated splenocytes from individual fish were added to the wells in triplicate at a concentration of 1 × 10^5^ cells per well. After 72 h of incubation at 20°C, cells were washed away 5 times with PBS and plates were blocked again with 2% BSA in PBS for 1 h at room temperature. After blocking, biotinylated anti-trout IgM mAb (clone 4C10) was added to the plates and incubated at 1 μg/ml for 1 h at room temperature. Following additional washing steps (5 times in PBS) the plates were developed using streptavidin-HRP (Thermo Scientific) for 1 h at room temperature, washed again with PBS and incubated with 3-amino 9-ethylcarbazole (Sigma Aldrich) for 30 min at room temperature in the dark. Substrate reaction was stopped by washing the plates with tap water. Once the membranes had dried, they were digitally scanned and spot counts determined by the ImmunoSpot Series 45 Micro ELISPOT Analyzer.

### Cell proliferation assay

A BrdU Flow Kit (Becton Dickinson) was used to measure the specific proliferation of IgM^+^ cells following the manufacturer's instructions. Splenocytes at a concentration of 2 × 10^6^ cells/ml were incubated for 3 days at 20°C with different stimuli (rIL-4/13A, rIL-4/13B2 or LPS as a positive control). Bromodeoxyuridine (BrdU, 10 μM) was then added to the cultures and cells were incubated for an additional 24 h. Afterwards the cells were collected and stained with a phycoerythrin (PE) conjugated anti-trout IgM mAb (1.14), then fixed and permeabilized with Cytofix/Cytoperm Buffer for 15 min on ice. Next the cells were incubated with Cytoperm Permeabilization Buffer Plus for 10 min on ice and re-fixed with Cytofix/Cytoperm Buffer for 5 min at room temperature. Cells were then incubated with DNase (30 μg/10^6^ cells) for 1 h at 37°C, to expose the incorporated BrdU. Finally, they were stained with a FITC conjugated anti-BrdU antibody for 20 min at room temperature and analysed by flow cytometry (BD FACSCalibur, BD Biosciences).

### Cell sorting

Splenocytes from individual fish were dispensed into 24-well plates at a density of 1 × 10^6^ cells/ml and incubated with rIL-4/13A, rIL-4/13B2 or LPS as a reference. Non-stimulated controls were also included. Cells were then incubated at 20°C for 24 h. The leucocytes were then collected from the wells by gentle scrapping, resuspended in PBS and incubated for 30 min on ice with a PE conjugated anti-trout IgM mAb (1.14) [74]. Following two washing steps, cells were resuspended in PBS and IgM^+^ cells were isolated by FACS sorting in a BD FACSAria III (BD Biosciences), using first their FSC/SSC profiles (to exclude the granulocyte gate) and then on the basis of the fluorescence emitted by the anti-trout IgM mAb [102]. IgM^+^ and IgM^−^ cells were then collected in different tubes for RNA isolation. Expression of IgM and the absence of T cell markers (CD3 and TCR) was verified by PCR in selected samples to confirm effective sorting.

### Statistical analysis

The data were statistically analyzed using the SPSS Statistics package 22 (SPSS Inc., Chicago, Illinois). The analysis of real-time PCR data was as described previously [29, 57]. One way-analysis of variance (ANOVA) and the LSD post hoc test were used to analyse expression data derived from the infection studies, with *p* < 0.05 between treatment and control groups considered significant. For the tissue distribution of expression and *in vitro* experiments that consisted of sample sets from individual fish, a Paired-Samples *T*-test was applied.

## SUPPLEMENTARY FIGURES AND TABLES



## Abbreviations

AMP: antimicrobial peptide
APP: acute phase protein
CDS: coding sequence
ERM: enteric redmouth disease
EST: expressed sequence tag
HK: head kidney
Ig: immunoglobulin
IL: interleukin
ILC2: type-2 innate lymphoid cells
i.p.: intraperitoneal
PAMPs: pathogen-associated molecular patterns
PGN: peptidoglycan
PHA: phytohaemagglutinin
PKD: proliferative kidney disease
Poly IC: polyinosinic-polycytidylic acid
RACE: rapid amplification of cDNA ends
STAT: signal transducer and activator of transcription
Th: T-helper
UTR: untranslated region
VHSV: viral haemorrhagic septicaemia virus
WGD: whole genome duplication
WGS: whole genome shotgun sequence
γC: common-γ chain
