# Supplementary material for: First in-depth analysis of the novel Th2-type cytokines in salmonid fish reveals distinct patterns of expression and modulation but overlapping bioactivities
Source: Oncotarget. 2016 Feb 9;7(10):10917–46. doi: 10.18632/oncotarget.7295 (PMC4905449; doi:10.18632/oncotarget.7295)
Supplement: Supplementary file 1 [file oncotarget-07-10917-s001.pdf]

First in-depth analysis of the novel Th2-type cytokines in salmonid fish reveals distinct patterns of expression and modulation but overlapping bioactivities

Supplementary Material

Table of contents

Fig. S1. Nucleotide and deduced amino acid sequences of trout IL-4/13A ………………………….. Page 2

Fig. S2. Nucleotide and deduced amino acid sequences of salmon IL-4/13A ………………..……….. Page 2

Fig. S3. Identification of a putative IL-4/13 pseudogene ……………………………………….….. Page 3

Fig. S4. Nucleotide and deduced amino acid sequences of salmon IL-4/13B1 …………………….. Page 3

Fig. S5. Nucleotide and deduced amino acid sequences of salmon IL-4/13B2 …………………….. Page 4

Fig. S6. Nucleotide and deduced amino acid sequences of trout IL-4/13B1 ……………………….. Page 4

Fig. S7. Nucleotide and deduced amino acid sequences of trout IL-4/13B2 ……………………….. Page 5

Fig. S8. Comparison of salmonid IL-4/13 protein sequences …………………………………….. Page 5

Fig. S9. Phylogenetic tree analysis …………………………..………………………………………Page 6

Fig. S10. Prediction of disulfide bonding and cysteine connectivity ……………………………….. Page7,8

Fig. S11. Dose responses of trout rIL-4/13A and rIL-4/13B2………………...……………….……... Page 9

Fig. S12. Effects of rIL-4/13 isoforms on IgM+ B cells from spleen ………………………….……… Page 10

Table S1. Identity (Top right)/similarity (Bottom left) analysis ………………………….……..…….. Page 11

Table S2. Primers used for expression analysis by real-time PCR……………………….……..….. Page 12

Rainbow trout IL-4/13A

```
  1  ACATTTGGCATTAAATTATTACCTGCTGCACCTCAACTTCTCTGAAGTACAAGGACTTCTTTAGTACTACTTAGCTTTGGCAAATCAACC

  1            M  K  T  A  I  L  L  L  S  V  A  M  V  L  F  D  S  G  T  L  P  A  N  A  A  A  H
 91  CAACCAAAGATGAAGACGGCGATCCTTCTCCTCTCTGTTGCGATGGTGCTATTTGACTCTGGAACTCTGCCTGCCAACGCTGCAGCGCAC

 28   R  Q  H  R  S  H  N  S  N  L  E  A  I  I  S  M  A  G  Q  Y  T  H  T  L  S  E  E  L  L  E
181  CGGCAGCATAGAAGTCACAATTCCAATTTAGAGGCAATTATTAGCATGGCAGGACAATACACACACACTCTGTCAGAGGAACTTCTGGAA

 58   T  L  L  I  D  V  T  H  L  T  A  T  T  T  K  C  K  E  F  F  C  E  A  E  T  I  L  A  S  V
271  ACATTACTCATAGATGTAACACATTTGACTGCGACCACCACAAAGTGCAAGGAGTTCTTCTGCGAAGCTGAGACAATCTTGGCCTCCGTG

 88   K  N  D  A  F  G  E  E  G  K  I  V  R  H  L  R  V  Y  N  K  L  Q  K  C  T  V  V  K  S  Q
361  AAAAATGACGCGTTTGGTGAGGAGGGAAAGATTGTCCGACATCTGAGGGTTTACAACAAACTTCAGAAGTGCACCGTTGTGAAGAGCCAA

118   D  Q  V  E  G  N  Q  V  E  L  R  R  L  L  L  D  L  K  E  C  G  Q  K  I  N  S  K  P  -
451  GACCAGGTGGAGGGCAACCAGGTAGAGCTGAGGAGACTTCTCCTCGACCTAAAAGAATGTGGCCAAAAAATCAACTCCAAACCATGAGAT
541  CTACAACATCTCAACACAATGCTAATCTGCTTATTTATTGTTTGTTGACCGTTTTATTTAAGTTAAAGTGGCGTTAAATGACTTTACCAA
631  GGGAAGTATTTTAATTATATAATGTTAATCTTTTTTCTTTTTCTTTTACCTGAGTAGCATATTGATAATCTAATGCACACCTGTTAAATA
721  ATATGCATAATTTTATATTACAATATTTGTACTGAAATAAAGGTTTTTCATGGCAAAAAAAAAAAAAAAAAAAA
```

Fig. S1 Nucleotide and deduced amino acid sequences of trout IL-4/13A. The cDNA sequence was obtained by 3’- and 5’-RACE. The in-frame stop codon in the 5’-UTR, and the start and stop codons for translation are highlighted in red. Putative mRNA instability motifs (ATTTA) and the potential polyadenylation signal in the 3’-UTR are in bold and boxed. Intron positions are indicated by arrows. A predicted signal peptide is highlighted in green.

Atlantic salmon IL-4/13A

```
  1  tcctcacatttggctttaaattattacctgctagcacctcaacttctccgaagtacaaggactttctttagtactacttagcgttggcAAA

  1                  M  K  S  V  I  L  L  S  V  A  M  V  L  F  N  S  G  T  L  P  A  N  A  A  A
 91  TAAACCCAACCAAAGATGAAGTCGGTGATCCTTCTCTCTGTTGCGATGGTGCTATTTAACTCTGGAACTCTGCCTGCCAACGCTGCAGCG

 26   Y  R  Q  H  K  N  H  H  S  N  L  E  A  I  I  S  M  A  E  Q  Y  T  H  N  L  T  E  D  L  L
181  TACCGGCAGCATAAAAATCACCATTCCAATTTAGAGGCAATTATTAGCATGGCGGAACAATACACACACAATCTGACAGAGGATCTTCTG

 56   R  T  L  L  I  D  V  T  H  L  T  G  T  T  T  K  C  K  E  F  F  C  E  A  E  K  I  L  A  S
271  AGAACATTACTCATAGATGTTACACATTTGACTGGGACCACCACAAAATGCAAGGAGTTCTTCTGCGAAGCTGAGAAAATCTTGGCCTCC

 86   V  K  N  A  T  F  G  N  I  V  R  H  L  R  V  Y  N  K  H  Q  Y  C  I  V  V  K  S  Q  D  N
361  GTGAAAAATGCCACGTTTGGAAATATTGTCCGACATCTGAGGGTTTACAACAAACATCAATACTGCATCGTTGTGAAGAGCCAAGACAAC

116   Q  A  E  G  N  Q  V  E  L  R  R  L  L  L  D  L  K  E  C  G  Q  K  I  N  S  K  P  -
451  CAGGCGGAGGGCAACCAGGTGGAGCTGAGGAGACTTCTCCTCGACTTAAAAGAATGTGGCCAAAAAATCAACTCCAAACCATGATCTACA

541  CCAACTCCACACAATGCTAatctgcttatttatttgttgacagttttatttaagtaagttagtggcattaaatgactttaccaagggcag

631  tattttaattatataatgttaaatctttttttttattttacctgagttgcatattgatgatctaatgcacacctgttaacaatgtgcataa

721  ttttatttgtactaaaataaaaaaaaatcataaataaattttaaagttttgtttttaa
```

Fig. S2 Nucleotide and deduced amino acid sequences of salmon IL-4/13A. The nucleotide sequence in upper case is from AB574339 and in lower case from WGS contig AGKD03034860. The in-frame stop codon in the 5’-UTR, the start and stop codons for translation are highlighted in red. Putative mRNA instability motifs (ATTTA) and two potential polyadenylation signals in the 3’-UTR are in bold and boxed. Intron positions are indicated by arrows. A predicted signal peptide is highlighted in green. Potential *N*-glycosylation sites are in purple.

Atlantic salmon IL-4/13A pseudogene

A.

```
aaaaacgagctgagtcttttCAGAAAAACTGCACCATTGTGGAGAAAAGCCAAGGCAAC
                      Q  K  N  C  T  I  V  E  K  S  Q  G  N
CAGGTGAAGCTCAGGAGACTACTCAACAACCTAAACGAATGTGGCCAGAAAATCTGGTAA
 Q  V  K  L  R  R  L  L  N  N  L  N  E  C  G  Q  K  I  W  -
cacctgttaacaatgtgaaaattgtgataaacaatatttttacaaaaataatgtcatttg
ctttcaactttcaaatctagcagaaatgttttcaatgacatacattaattgtcaatggac
aggttagactggtccttcccactgagatcgcaaaaaaattattatcctggccttcatcca
cagtagctgaaatctaaatctaaatagaagactacggattgcaaaacttactgatatctc
actcacatacgcttgaaatcactattactttgtgtttgctagccatgggtctgggtctac
ctgtagtgggccttactgtaaatgctggggctgaagagcttttggtgtgaacttgctttg
ccactctcctacttccccggacaatgtttaagttcaagttttgatttgtcacatgacaat
gatacacatggtatatccaccgtttaacaaaatgcttacttgcaggttccttctcgacaa
tgcaacaacaataagaaatattaataggtaagaatacaaacataacgtcaatggacaatg
taaagatctctcagtgacttgatgcaaggtgagctctgagtagaggccatacagttggag
ctttactattggctagcagccgtccatcgctgtatgtgctaccttattactaaataaaac
cagaatctccctcccttt
```

B.

```
                                                                  Identities (%)
                                                               Salmon p  Salmon A  Trout A
Salmon IL-4/13p    QKNCTIVEKSQ-----GNQVKLRRLLNNLNECGQKIW        100       48.7      61.5
Salmon IL-4/13A    QY-CIVV-KSQDNQAEGNQVELRRLLLDLKECGQKINSKP     48.7      100       89.5
Trout IL-4/13A     QK-CTVV-KSQD-QVEGNQVELRRLLLDLKECGQKINSKP     61.5      89.5      100
                   *: * :* ***      ****:***** :*:******
```

Fig. S3 Identification of a putative IL-4/13 pseudogene. (A) The nucleotide sequence of the last exon of a putative IL-4/13 pseudogene in Atlantic salmon. The sequence is from WGS contig AGKD03032511 (reverse complement from 263373 to 264170). The coding region and its translation is shown in capital letters. A potential *N*-glycosylation site is in purple. The potential polyadenylation signal is in bold and boxed. A canonical intron acceptor site (AG) is missing at the intron junction and mRNA instability motifs (ATTTA) are missing in the putative 3'-UTR. **(B)** Comparison of amino acid sequences encoded by exon 4 of salmonid IL-4/13A genes and the putative salmon IL-4/13 pseudogene.

Atlantic salmon IL-4/13B1

```
  1                                                                                                    M  K
  1  AGTCACATTTGAACCTTCTTCACCCCGAGACAGTATCAAGTCATAGCCTGCTACTACTACTACTACAACAACTACTGTAAAGCCATGAAG

  3   T  L  A  L  L  F  S  F  A  F  V  L  V  F  T  A  P  T  K  T  S  D  E  H  H  L  L  R  R  I
 91  ACTCTTGCTCTCCTCTTCTCCTTTGCATTTGTGCTGGTCTTCACTGCGCCCACTAAGACATCTGATGAGCATCATCTACTGAGGAGGATC

 33   M  I  K  A  N  K  T  R  D  E  G  P  E  A  L  L  D  S  L  V  P  A  Q  F  N  Q  V  R  C  K
181  ATGATCAAGGCTAACAAGACCCGCGATGAAGGCCCGGAGGCCCTTTTGGACAGTCTAGTGCCAGCGCAATTCAATCAAGTTCGCTGCAAG

 63   E  H  G  P  K  D  F  C  I  A  E  K  I  L  S  N  I  N  E  E  Q  Y  G  T  S  D  N  N  I  G
271  GAACACGGGCCTAAAGATTTCTGCATAGCGGAGAAGATTCTGTCTAACATAAACGAAGAGCAGTACGGGACTTCAGACAACAACATTGGG

 93   T  I  S  R  E  L  K  Q  Y  N  M  L  H  P  S  N  C  T  V  K  N  N  V  D  E  E  Q  L  R  A
361  ACAATCAGCAGAGAGCTGAAGCAGTACAATATGCTTCACCCTTCCAACTGCACAGTGAAGAATAACGTTGACGAGGAACAGCTCCGCGCT

123   L  L  M  N  L  Y  D  C  A  Q  I  I  Y  A  R  P  H  P  V  T  H  H  K  T  T  P  A  L  -
451  CTCCTGATGAACCTCTACGACTGTGCCCAAATCATCTATGCCCGTCCCCACCCTGTCACTCATCACAAAACGACCCCAGCACTTTGAGGG

541  GACTGATGGGAACTCTGTCGATCGTGCATAGTCCAATAACACATATGATTATTTATTGAATTGTATTTAAATGTACTATGACATTTATTT

631  TTGTACTATTTATGAATAAATTAACTAATTGCTAAAAACAAAAAAAAAAAAAAAAAAAAAAAAAAA
```

Fig. S4 Nucleotide and deduced amino acid sequences of salmon IL-4/13B1. The cDNA sequence was obtained by 3'- and 5'-RACE. The in-frame stop codon in the 5'-UTR, the start and stop codons for translation are highlighted in red. Putative mRNA instability motifs (ATTTA) and the polyadenylation signal in the 3'-UTR are in bold and boxed. Intron positions are indicated by arrows. A predicted signal peptide is highlighted in green. Potential *N*-glycosylation sites are in purple.

Atlantic salmon IL-4/13B2

```
  1                                                                           M  K  T  V  T  L
  2  CCTTCTTCACCCCGAGGGCTGAGACTGTATCTAGTTATAGCCTGCTACCACTACTACTACTACTGTAAAGACATGAAGACTGTCACACTC

  7  L  F  S  F  A  F  V  L  V  F  T  A  P  T  D  E  T  H  L  L  L  T  I  I  D  E  A  N  K  I
 92  CTCTTCTCCTTTGCATTTGTGCTGGTCTTCACTGCGCCCACTGATGAGACTCATCTATTGCTTACGATCATCGACGAGGCTAACAAGATC

 37  L  N  G  G  L  E  F  Q  A  L  V  D  S  L  V  P  A  G  F  D  Q  D  R  C  R  E  N  G  P  E
182  CTCAATGGAGGTTTGGAGTTTCAGGCCCTTGTGGACAGTCTAGTGCCAGCGGGATTCGATCAAGATCGCTGCAGGGAAAACGGGCCTGAA

 67  D  F  C  I  A  E  T  I  L  S  A  I  N  H  A  Q  Y  R  S  P  L  N  N  I  S  K  I  S  R  V
272  GATTTCTGCATAGCTGAGACTATTCTGTCTGCCATAAACCATGCGCAGTACAGGAGTCCATTAAACAACATATCTAAAATCAGTAGAGTG

 97  L  K  L  Y  N  K  F  H  P  T  N  C  T  V  K  E  N  G  D  E  E  Q  L  H  D  L  L  T  N  L
362  CTGAAGCTGTACAATAAGTTTCATCCAACCAACTGCACTGTGAAGGAGAACGGTGATGAGGAACAGCTCCACGATCTCCTGACAAACCTC

127  F  N  C  A  Q  A  I  Y  S  C  P  S  P  C  R  S  S  Q  T  T  L  -
452  TTCAACTGTGCCCAGGCCATTTACTCTTGTCCCTCACCCTGCCGCTCATCACAAACAACCCTATAGCTTTGCTGAGAGGACTGATGGAAA

542  CTCTGTTGACTCTGCATAGTCCAGTACCTCATATTGTTATTGTATTGTATTATTTAAATGTATTATGGTATTTACTTTTGTACTATTTAT

632  GAATAAATTAACTTGTAAAAGTGGCAAAAAAAAAAAAAAAAAAAAAAAAAA
```

Fig. S5 Nucleotide and deduced amino acid sequences of salmon IL-4/13B2. The cDNA sequence was obtained by 3'- and 5'-RACE. The in-frame stop codon in the 5'-UTR, and the start and stop codons for translation are highlighted in red. Putative mRNA instability motifs (ATTTA) and the polyadenylation signal in the 3'-UTR are in bold and boxed. Intron positions are indicated by arrows. A predicted signal peptide is highlighted in green. Potential *N*-glycosylation sites are in purple.

Rainbow trout IL-4/13B1

```
  1                                                                      M  K  T  L  A  L  L  F
  1  TTGAACCTTCTTCACCCCGAGACAGTATCAAGTCACAGCCTGCTACTACTTCTACTACTGTAAAGCCATGAAGACTCTTGCTCTCCTCTTC

  9  S  F  A  F  V  L  V  F  T  A  P  T  K  T  P  D  E  I  H  L  L  Q  R  I  M  K  E  A  N  K
 92  TCCTTTGCATTTGTGCTGGTCTTCACTGCGCCCACTAAGACACCTGATGAGATTCATCTACTGCAGAGGATCATGAAAGAGGCTAACAAG

 39  T  L  K  E  G  P  E  A  L  L  N  H  L  V  P  A  E  F  K  Q  D  R  C  K  S  H  G  P  K  D
182  ACGCTCAAGGAAGGCCCGGAGGCCCTTTTGAACCATCTAGTGCCAGCGGAATTCAAGCAAGATCGCTGCAAGTCACACGGGCCTAAAGAT

 69  F  C  I  A  E  T  I  L  S  N  I  N  K  T  Q  Y  G  T  S  D  N  N  I  E  T  I  S  R  V  L
272  TTCTGCATAGCGGAGACGATTCTGTCTAACATAAACAAAACGCAGTACGGGACTTCAGACAACAACATAGAGACAATCAGCAGAGTGCTG

 99  E  Q  Y  N  K  L  H  P  S  N  C  T  M  K  T  N  G  D  E  E  Q  L  R  G  L  L  M  N  L  Y
362  GAGCAGTACAATAAGCTTCACCCTTCCAACTGCACTATGAAGACGAATGGAGACGAGGAACAGCTCCGTGGTCTCCTGATGAACCTCTAC

129  N  C  A  Q  A  I  Y  S  R  P  H  P  V  T  H  H  K  T  T  P  A  L  -
452  AACTGTGCCCAAGCCATCTATTCCCGTCCCCACCCTGTCACTCATCACAAAACGACCCCAGCACTTTGAGGGGACCTCTGTCGATCGTGC

542  ATAGTCCAATaacacaaattatttttattgaattgtatttaaatgtactatgacatttatttttgtactatttatgaataaattaacta

632  attggtaaaaact
```

Fig. S6 Nucleotide and deduced amino acid sequences of trout IL-4/13B1. The cDNA sequence in upper case was amplified using primers tIL-4b1F1 and R1 (primer binding sites are shaded). The nucleotide sequence in lower case from WGS contig CCAF010144013. The in-frame stop codon in the 5'-UTR, and the start and stop codons for translation are highlighted in red. Putative mRNA instability motifs (ATTTA) and two potential polyadenylation signals in the 3'-UTR are in bold and boxed. Intron positions are indicated by arrows. A predicted signal peptide is highlighted in green. Potential *N*-glycosylation sites are in purple.

Rainbow trout IL-4/13B2

```
  1                                                                                              M  K  T  V
  1 ACCTTCTTCACCCCGAGGGCTGACTGTATCTAGTCATAGCCTGCTGCTACTACTACCATTACTACTACTGCTGTAAAGCCATGAAGACTGTC

  5   A  L  L  F  S  F  A  F  V  L  V  F  T  A  P  T  K  T  S  D  E  T  H  L  L  R  M  I  I  D
 93   GCTCTCCTCTTCTCCTTTGCATTTGTGCTGGTCTTCACTGCGCCCACTAAGACATCTGATGAGACTCATCTATTGCGTATGATCATCGAC

 35   E  V  N  K  I  L  K  G  G  L  E  F  Q  A  L  L  D  S  L  V  P  A  G  F  D  Q  D  R  C  R
183   GAGGTTAACAAGATCCTCAAAGGAGGTCTGGAGTTTCAGGCCCTTTTGGACAGTCTAGTGCCGGCGGGATTCGATCAAGATCGCTGCAGG

 65   A  N  G  P  K  D  F  C  I  A  E  T  I  L  S  A  I  S  H  T  R  H  R  I  P  F  N  N  I  S
273   GCAAACGGGCCTAAAGATTTCTGCATAGCTGAGACTATTCTGTCTGCCATAAGCCATACGAGGCACAGGATTCCATTTAACAACATATCT

 95   K  I  S  R  V  L  K  Q  Y  N  K  F  H  P  T  N  C  N  V  K  E  N  G  D  E  E  Q  L  H  D
363   AAAATCAGTAGAGTGCTGAAGCAGTACAATAAGTTTCATCCAACCAACTGCAATGTGAAGGAGAACGGTGATGAGGAACAGCTCCACGAT

125   L  L  T  N  L  V  N  C  A  K  V  I  Y  S  R  P  P  P  C  R  S  S  Q  T  T  L  E  L  C  -
453   CTCCTGACAAACCTTGTCAACTGTGCCAAGGTCATCTACTCTCGTCCCCCTCCCTGCCGGTCTTCACAAACAACCCTAGAGCTTTGCTGA

543   GAGGACTGATGGAAACGCTGTCaactctgcatagtccagtacctcgtattgttattta ttgtattatttaaatgtattatgacagttatt

633   tttgtactatttatgaataaattaacttgtaaaagtggcaa
```

Fig. S7 Nucleotide and deduced amino acid sequences of trout IL-4/13B2. The cDNA sequence in upper case was amplified using primers tIL-4b2F1 and R1 (primer binding sites are shaded). The nucleotide sequence in lower case is from WGS contig CCAF010096158. The in-frame stop codon in the 5'-UTR, and the start and stop codons for translation are highlighted in red. Putative mRNA instability motifs (ATTTA) and two potential polyadenylation signals in the 3'-UTR are in bold and boxed. Intron positions are indicated by arrows. A predicted signal peptide is highlighted in green. Potential *N*-glycosylation sites are in purple.

```
Salmon-A   MKS-VILLSVAMVLFNSGTLPANAAAYRQHKNHHSNLEAIISMAEQYTHNLTEDLLRTLLIDVTHLTGTTTKCKE----FFCEAEKIL
Trout-A    MKTAILLLSVAMVLFDSGTLPANAAAHRQHRSHNSNLEAIISMAGQYTHTLSEELLETLLIDVTHLTATTTKCKE----FFCEAETIL
Salmon-B1  MKTLALLFSFAFVLVFT--------APTKTSDEHHLLRRIMIKANKTRDEGPE----ALLDSLVPAQFNQVRCKEHGPKDFCIAEKIL
Trout-B1   MKTLALLFSFAFVLVFT--------APTKTPDEIHLLQRIMKEANKTLKEGPE----ALLNHLVPAEFKQDRCKSHGPKDFCIAETIL
Salmon-B2  MKTVTLLFSFAFVLVFT--------APT---DETHLLLTIIDEANKILNGGLE--FQALVDSLVPAGFDQDRCRENGPEDFCIAETIL
Trout-B2   MKTVALLFSFAFVLVFT--------APTKTSDETHLLRMIIDEVNKILKGGLE--FQALLDSLVPAGFDQDRCRANGPKDFCIAETIL
            Signal peptide                                                              C         C

Salmon-A    AS-VKNATFG-------NIVRHLRVYNKH--QYCIVVKSQDNQAEGNQVELRRLLLDLKECGQKINSKP------------- 142
Trout-A     AS-VKNDAFGE----EGKIVRHLRVYNKL--QKCTVVKSQD-QVEGNQVELRRLLLDLKECGQKINSKP------------- 145
Salmon-B1   SNGINEEQYGTSDNNIGTISRELKQYNMLHPSNCTVKN------NVDEEQLRALLMNLYDCAQIIYARPHPVTHHKTTPAL- 150
Trout-B1    SNGINKTQYGTSDNNIETISRVLEQYNKLHPSNCTMKT------NGDEEQLRGLLMNLYNCAQAIYSRPHPVTHHKTTPAL- 150
Salmon-B2   SAGINHAQYRSPLNNISKISRVLKLYNKFHPTNCTVKE------NGDEEQLHDLLTNLFNCAQAIYSCPSPCRSSQTTL--- 147
Trout-B2    SAGISHTRHRIPFNNISKISRVLKQYNKFHPTNCNVKE------NGDEEQLHDLLTNLVNCAKVIYSRPPPCRSSQTTLELC 153
                                              C                         C
```

Fig. S8 Comparison of salmonid IL-4/13 protein sequences. The multiple alignment was produced using ClustalW. The predicted signal peptides are underlined and potential N-glycosylation sites are boxed. The four conserved cysteine residues are indicated below the alignment.

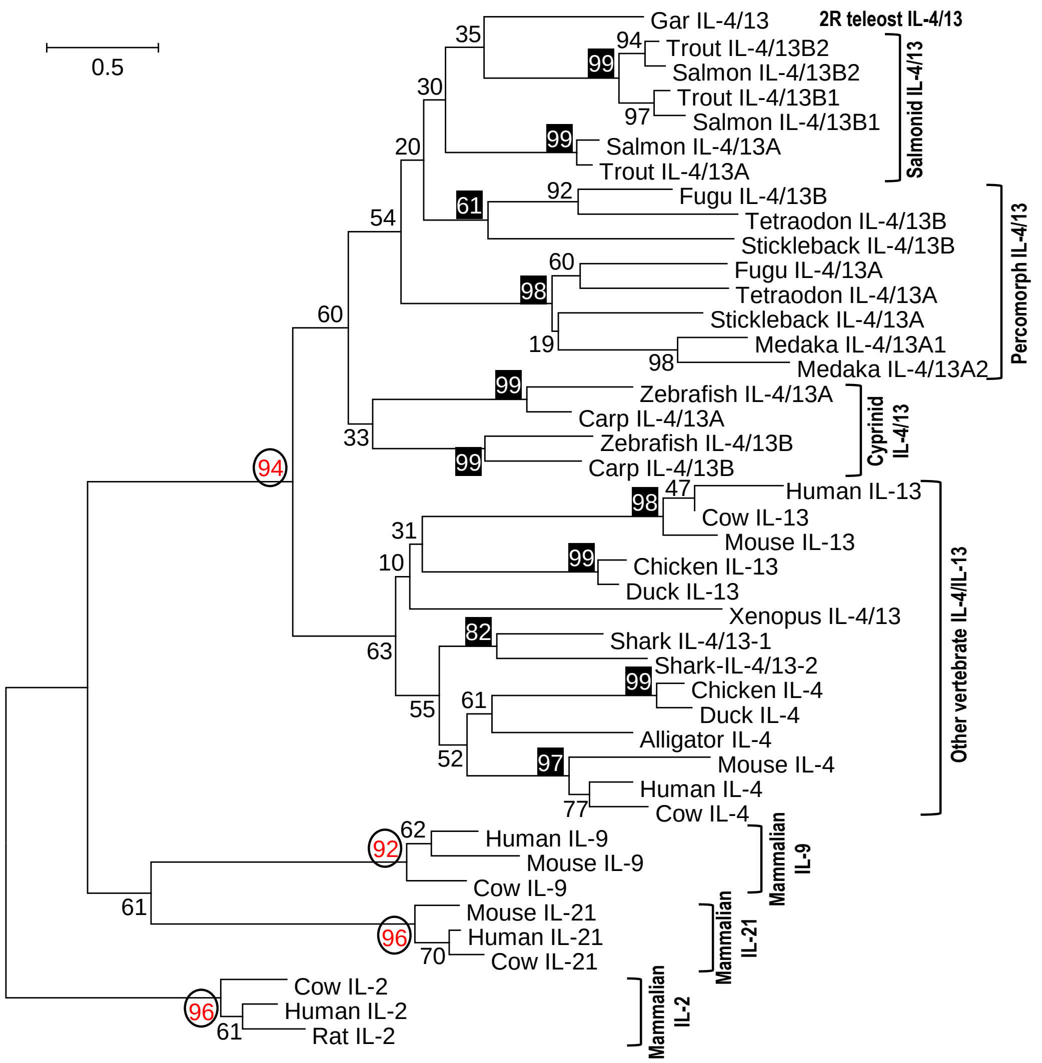

Fig. S9. Phylogenetic tree analysis of fish IL-4/13 with tetrapod IL-4/IL-13 molecules and other closely related γ-chain cytokines IL-2, IL-9 and IL-21. The phylogenetic tree was constructed using amino acid multiple alignments and the neighbour-joining method within the MEGA6 program [44]. The percentage of replicate trees in which the associated taxa clustered together in the bootstrap test (10,000 replicates) was shown next to the branches. The evolutionary distances were computed using the JTT matrix-based method with all ambiguous positions removed for each sequence pair. Spotted gar IL-4/13 was predicted from chromosome LG6. The amino acid sequences of IL-4/13A and B molecules of fugu, tetraodon, stickleback and medaka were extracted from Ohtani et al. [32]. Accession numbers for salmonid molecules are provided in Table 2. The accession numbers of other molecules are B3IWZ9 (zebrafish IL-4/13A), I0IV50 (carp IL-4/13A), D1YSM1 (zebrafish IL-4/13B), H1AFL4 (carp IL-4/13B), XP_007900235 (shark IL-4/13-1), XP_006023629 (shark IL-4/13-2), A9JPI4 (frog IL-4), XP_006023629 (alligator IL-4), P05112 (human IL-4), P07750 (mouse IL-4), P30367 (cow IL-4), C4PAF0 (chicken IL-4), G1N9Y7 (duck IL-4), P35225 (human IL-13), P20109 (mouse IL-13), Q9XSV9 (cow IL-13), C4PA62 (chicken IL-13), A2VBZ3 (duck IL-13), P60568 (human IL-2), P17108 (rat IL-2), P05016 (cow IL-2), P15248 (human IL-9), P15247 (mouse IL-9), E1BF10 (cow IL-9), Q9HBE4 (human IL-21), Q9ES17 (mouse IL-21) and Q76LU5 (cow IL-21). The bootstrapping values that support lineage-specific groupings are highlighted with a black background.

A. Salmon IL-4/13A

```
                                                         +----+
                                                         |    |
         .........10........20........30........40........50........60........70........
AA       AAYRQHKNHHSNLEAIISMAEQYTHNLTEDLLRTLLIDVTHLTGTTTKCKEFFCEAEKILASVKNATFGNIVRHLRVYN
DB_state                                                 1    1
DB_conf                                                  4    5
             +--------------------------+
             |                          |
         80........90........100.......110.......         DB_bond   bond(49,54)
AA       KHQYCIVVKSQDNQAEGNQVELRRLLLDLKECGQKINSKP         DB_bond   bond(84,111)
DB_state     1                          1                 Conn_conf 0.620818
DB_conf      5                          5
```

B. Salmon IL-4/13B

```
                                                    +--------+
                                                    |        |
         .........10........20........30........40........50........60........70........
AA       APTKTSDEHHLLRRIMIKANKTRDEGPEALLDSLVPAQFNQVRCKEHGPKDFCIAEKILSNINEEQYGTSDNNIGTISR
DB_state                                            1        1
DB_conf                                             5        5
                     +--------------------+
                     |                    |
         80........90........100.......110.......120.......130.    DB_bond   bond(44,53)
AA       ELKQYNMLHPSNCTVKNNVDEEQLRALLMNLYDCAQIIYARPHPVTHHKTTPAL    DB_bond   bond(92,113)
DB_state             1                    1                         Conn_conf 0.679736
DB_conf              5                    5
```

C. Carp IL-4/14A

```
                                                  +-----+
                                                  |     |
         .........10........20........30........40........50........60........70........
AA       TPVENDDKILLGELIDELNREVKRFSNDITEIFLAELEMNGCKGEFFCQAEHELKEKVSGLSGAKFEHFRTDKKLMRNL
DB_state                                          1     1
DB_conf                                           4     4
                   +--------------------+
                   |                    |
         80........90........100.......110.......120      DB_bond   bond(42,48)
AA       NGYNKRHVKTCKPADKDQEILLHVFLRNLLTCAKRVYSQPK        DB_bond   bond(90,111)
DB_state           1                    1                 Conn_conf 0.650489
DB_conf            4                    5
```

D. Carp IL-4/13B

```
                                                 +-----+                               +
                                                 |     |                               |
         .........10........20........30........40........50........60........70........
AA       KHKAGQILLMEIIDDVKQILNQSSSTNLNQFVIDVFPPVGCSEKHICQAAMVMMNTELSHSMLHRRLFAYANYSGHLHC
DB_state                                         1     1                               1
DB_conf                                          4     4                               4
         -------------------+
                            |
         80........90........100.......110       DB_bond   bond(41,47)
AA       NVTASEEHRMDVFLEKIKDCCKAQYSKPLKQ         DB_bond   bond(79,99)
DB_state                    1                    Conn_conf 0.612333
DB_conf                     4
```

E. Spotted gar IL-4/13

```
                                                     +-----+
                                                     |     |
         .........10........20........30........40........50........60........70........
AA       APTSKMSEHKGRRVLEEMIKHLTQLNNTLSQKIKEKFVVDVKKTCEPTFFCQIQKALKSLNHTEFGTDGVLMRLLDEYD
DB_state                                             1     1
DB_conf                                              5     5
                     +--------------------+
                     |                    |
         80........90........100.......110.......120      DB_bond   bond(45,51)
AA       LSIKQMSHHKGNCKIDDNGDQVQLHALLNKMKDCIQKINSKPN      DB_bond   bond(92,113)
DB_state             1                    1               Conn_conf 0.647207
DB_conf              5                    5
```

Fig. S9, continnued.

F. Shark IL-4/13-2

```
                                  +---------------------+           +-------------------
                                  |                     |           |
         .........10........20........30........40........50........60........70........
AA       KVDPKKHILREITKTLGHMTQDKIECGATLIPDVFSDTKGQSASEIFCKAAEALVKIPPCESIKRHLKALRLNLAKEGE
DB_state                          1                     1           1
DB_conf                           3                     3           2
         ----+
             |
         80........90........100.......110      DB_bond   bond(26,48)
AA       QTLSCPVNKFSEIELTTFLRKLRKLSQQKYRQ       DB_bond   bond(60,84)
DB_state     1                                  Conn_conf 0.827884
DB_conf      4
```

G. Xenopus IL-4/13

```
                                     +------------------+         +---------------------
                                     |                  |         |
         .........10........20........30........40........50........60........70........
AA       NPVPSSKLQIAIEEIISELVNNKITHKKCFVPTPYDDEEEASVEEISCRAFKSLKHVCASERKNLRLLNASLITMFSEN
DB_state                             1                  1         1
DB_conf                              5                  5         5
         --+
           |
         80........90........100.......110....           DB_bond   bond(29,48)
AA       VECSINNDEQKDLISVIEDLLTFFRAQMRKLVMNPKH           DB_bond   bond(58,82)
DB_state   1                                             Conn_conf 0.644412
DB_conf    5
```

H. Chicken IL-4

```
                                    +---------------------+           +-----------------
                                    |                     |           |
         .........10........20........30........40........50........60........70........
AA       VPMLCLQLSVPLMESIRIVNDIQGEVSCVKMNVTDIFADNKTNNKTELLCKASTIVWESQHCHKNLQGLFLNMRQLLNA
DB_state                            1                     1           1
DB_conf                             3                     2           0
         --------+
                 |
         80........90........100.     DB_bond   bond(28,50)
AA       SSTSLKAPCPTAAGNTTSMEKFLA     DB_bond   bond(62,88)
DB_state         1                    Conn_conf 0.729865
DB_conf          3
```

I. Chicken IL-13

```
                                      +---------------------+           +---------------
                                      |                     |           |
         .........10........20........30........40........50........60........70........
AA       TPLAMNLSKLKLSDITQGIQKLNRGAQVPCNDTRVAQVAFKDRKLSEQELLCQAATVLDNMTDCKKDYEPLITSLKSLH
DB_state                              1                     1           1
DB_conf                               5                     5           6
         ----+
             |
         80........90........100.......110......         DB_bond   bond(30,52)
AA       GMTNCPPSTDNEIYLRNFLPALGNYTQALYRRISATAAN         DB_bond   bond(64,84)
DB_state     1                                           Conn_conf 0.642958
DB_conf      6
```

Keys:

AA amino acid sequence

DB_state predicted disulfide bonding state (1=disulfide bonded, 0=not disulfide bonded)

DB_conf confidence of disulfide bonding state prediction (0=low to 9=high)

DB_bond position in sequence of a pair of cysteines predicted to be forming a disulfide bridge.

Conn_conf confidence of connectivity assignment given the predicted disulfide bonding state (real value in [0,1])

Fig. S10 Prediction of disulfide bonding and cysteine connectivity in salmon IL-4/13A (A), salmon IL-4/13B, carp IL-4/13A (C), carp IL-4/13B (D), spotted gar IL-4/13 (E), shark IL-4/14-2 (F), frog IL-4/13 (G), chicken IL-4 (H) and chicken IL-13 (I). The disulfide bonds were predicted using the DISULFIND program (http://disulfind.dsi.unifi.it).

Reference:

Ceroni A, Passerini A, Vullo A, Frasconi P. 2006 DISULFIND: a disulfide bonding state and cysteine connectivity prediction server. Nucleic Acids Res. 34(Web Server issue):W177-81.

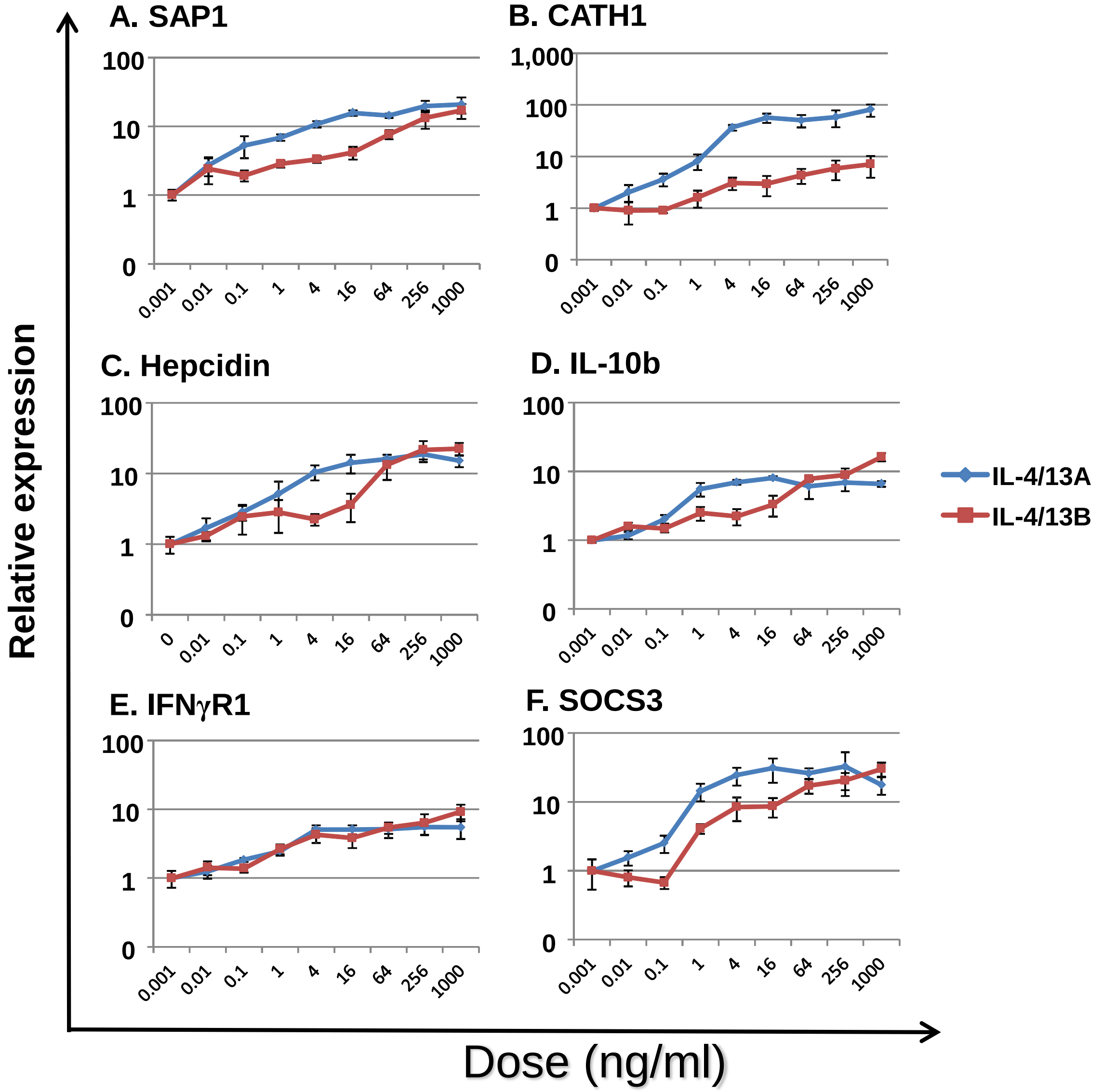

Fig. S11. Dose responses of trout rIL-4/13A and rIL-4/13B2. Freshly prepared HK cells were stimulated with rIL-4/13A or rIL-4/13B2 at 0.01, 0.1, 1, 4, 16, 64, 256, and 1000 ng/ml for 4 h. Gene expression of SAP1 (**A**), CATH1 (**B**), Hepcidin (**C**), IL-10b (**D**), IFNγR1 (**E**) and SOCS3 (**F**) was determined real-time PCR. The relative gene expression was calculated as the expression levels in stimulated cells normalized to that of un-stimulated controls. The means of cells from four fish are shown.

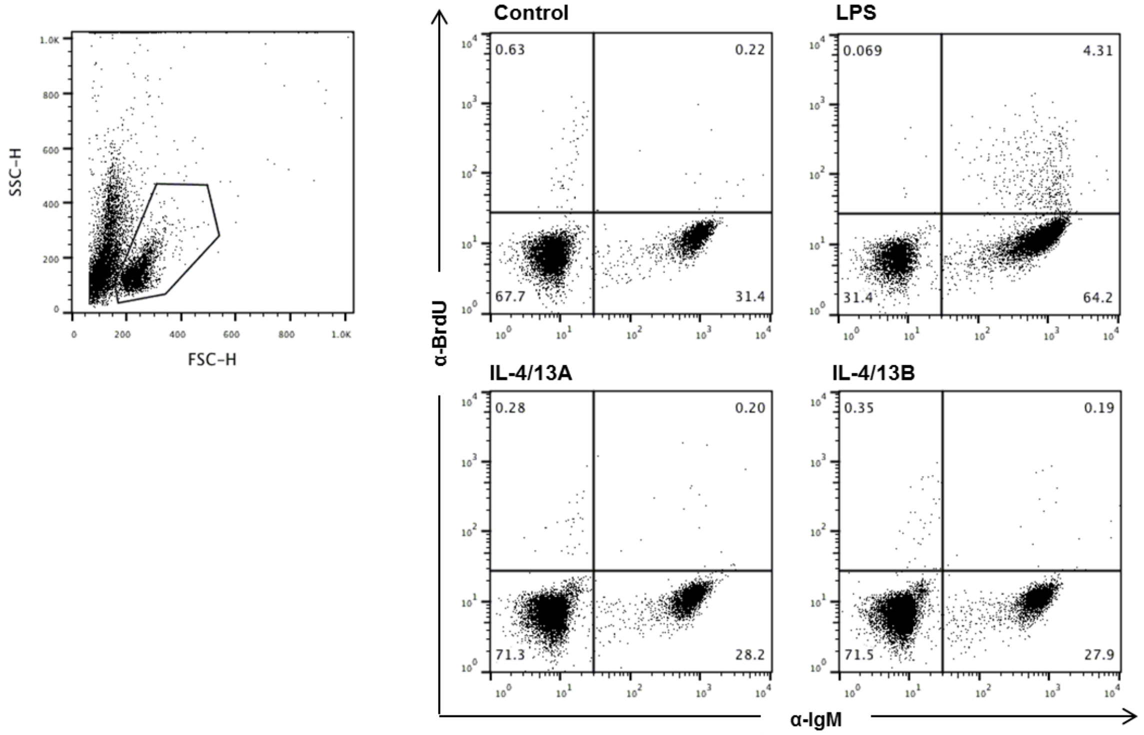

Fig. S12. Effects of rIL-4/13 isoforms on IgM$^+$ B cells from spleen. Spleen leucocytes (n=11) were incubated with/without rIL-4/13A, rIL-4/13B, or LPS as positive control for 3 days at 20ºC. The proliferating cells were then labeled with BrdU and incubated for a further 24 h. The proliferating (BrdU positive) cells and IgM$^+$ B cells was then determined by flow cytometry. A representative experiment of 11 performed is shown.

Table S1 Identity (Top right)/similarity (Bottom left) analysis of IL-4/13, IL-4, IL-13 and other IL-2 family cytokines

	Salmonid IL-4/13						Gar	Percomorph IL-4/13								Cyprinid IL-4/13				Shark IL-4/13				Chicken IL-4		Chicken IL-13		Mammalian IL-4			Mammalian IL-13			Mammalian IL-2			Mammalian IL-9			Mammalian IL-21			Mammalian IL-7			Mammalian IL-15		
Identities to salmonid IL-4/13							24.6	23.9								25.0				20.8		19.1		17.6		20.5		20.8			18.9			20.3			18.7			18.9			18.3			17.1		
	1	2	3	4	5	6	7	8	9	10	11	12	13	14	15	16	17	18	19	20	21	22	23	24	25	26	27	28	29	30	31	32	33	34	35	36	37	38	39	40	41	42	43	44	45	46	47	48
1. Salmon IL-4/13A		81.5	28.0	27.6	27.1	26.8	29.7	24.5	18.9	28.9	26.0	28.0	25.0	23.8	24.0	28.0	25.3	18.4	22.7	21.4	22.2	21.4	20.7	17.1	19.6	16.3	17.9	24.1	19.5	23.6	19.4	16.6	18.2	18.9	21.1	19.6	17.8	20.8	18.0	21.2	21.7	21.1	16.9	21.2	18.4	20.5	18.3	21.7
2. Trout IL-4/13A	89.0		28.7	29.3	28.5	26.3	29.6	27.6	21.0	30.7	27.9	30.1	23.3	24.7	24.0	28.7	26.6	19.7	20.9	22.2	22.1	21.7	18.8	17.4	19.9	19.4	20.6	26.2	21.9	24.0	19.4	19.2	21.1	18.4	20.2	17.9	15.8	21.6	20.5	18.6	17.3	24.8	17.3	21.0	16.6	17.9	19.2	17.0
3. Salmon IL-4/13B1	46.0	46.0		82.0	61.8	62.7	27.5	21.9	23.5	21.7	23.6	20.3	27.0	25.5	19.2	24.7	26.8	19.6	22.2	18.4	20.2	20.5	16.1	19.2	15.9	22.0	19.9	16.3	21.3	17.0	17.1	18.7	19.5	21.0	17.2	20.3	19.5	17.8	15.4	16.7	18.4	16.3	15.3	18.6	14.5	14.8	15.8	15.0
4. Trout IL-4/13B1	43.3	44.7	88.0		65.1	66.0	28.1	25.2	20.2	21.3	22.2	21.5	24.7	26.8	21.3	25.1	30.1	22.4	23.5	18.1	20.9	20.3	16.8	15.9	20.1	23.7	22.2	16.0	19.8	18.4	17.6	20.2	17.5	21.9	20.1	22.6	18.4	19.0	16.6	17.5	17.0	14.8	15.8	18.7	15.0	14.9	16.1	16.9
5. Salmon IL-4/13B2	46.3	46.9	72.7	74.7		82.4	24.5	22.7	21.7	24.4	21.7	21.0	21.5	28.7	23.5	27.1	31.4	24.0	24.7	22.6	21.9	19.2	18.5	18.8	14.3	22.4	22.4	20.7	21.1	24.3	18.9	21.6	15.9	22.2	20.7	20.5	23.6	17.0	15.6	17.8	16.4	17.4	16.9	22.0	18.8	16.0	19.8	15.6
6. Trout IL-4/13B2	42.5	43.8	72.5	75.8	86.3		25.8	22.4	20.5	23.6	24.5	21.7	25.3	28.8	22.9	25.0	31.8	27.3	24.8	17.6	22.4	15.1	19.5	15.5	17.1	19.2	19.6	21.6	20.0	18.3	19.7	20.9	18.8	21.8	21.6	19.7	17.7	19.9	20.7	22.9	19.4	20.0	19.7	22.6	20.5	15.1	18.9	14.4
7. Gar IL-4/13	52.1	55.2	46.7	49.3	45.6	45.1		17.2	24.2	24.7	26.4	26.1	21.6	24.8	23.8	27.4	28.0	20.5	23.5	18.6	18.3	23.1	24.3	15.2	18.2	22.0	21.4	24.7	17.5	22.1	21.3	14.4	20.9	21.2	22.9	23.2	18.6	19.9	20.6	20.1	25.5	24.2	17.7	24.1	19.8	16.9	17.5	18.9
8. Tetraodon IL-4/13A	41.5	44.8	34.7	39.3	39.5	36.6	46.5		33.6	32.7	27.8	27.5	24.2	22.0	18.5	18.9	23.6	23.0	23.6	26.5	23.3	21.3	23.7	22.1	22.9	20.5	22.6	21.7	20.8	20.3	21.3	23.5	20.7	18.1	18.7	20.7	19.6	14.7	15.9	16.7	18.2	16.4	16.7	15.5	16.8	15.3	21.3	19.6
9. Fugu IL-4/13A	40.8	42.1	37.3	37.3	36.7	37.9	46.5	50.0		25.5	29.3	29.3	21.0	20.8	19.6	17.3	19.0	20.9	20.0	20.9	22.9	19.4	12.7	20.9	19.7	20.3	21.4	19.4	17.9	21.2	20.0	21.1	19.0	15.8	19.4	15.2	18.2	23.7	17.9	20.7	20.1	22.4	15.8	21.4	19.6	17.6	17.6	14.9
10. Stickleback IL-4/13A	49.3	43.4	36.7	39.3	38.1	35.9	45.1	54.7	50.0		30.6	28.8	19.6	18.6	18.4	18.5	24.2	18.9	18.6	23.8	23.8	22.1	20.8	18.8	20.0	17.5	18.8	15.9	16.6	18.4	16.8	14.6	17.6	19.3	17.3	17.3	15.8	17.8	18.3	16.6	18.7	19.9	17.8	17.9	17.0	16.0	18.9	18.3
11. Medaka IL-4/13A1	42.3	47.6	39.3	34.0	42.9	39.2	47.2	48.9	49.3	51.8		45.1	17.9	19.2	20.5	21.7	23.0	21.6	17.7	17.9	23.3	18.4	25.0	18.8	18.2	20.1	18.6	21.7	21.4	21.6	21.1	28.3	22.5	17.8	17.7	19.9	21.4	18.9	20.0	22.7	22.4	21.0	16.2	19.3	18.7	16.6	17.1	16.1
12. Medaka IL-4/13A2	50.7	52.4	37.3	36.7	39.5	37.9	49.3	46.0	44.3	45.3	61.9		20.5	20.5	19.4	19.0	25.2	20.0	21.2	23.3	24.1	22.4	22.8	24.2	21.2	24.2	22.0	19.5	18.2	23.3	15.9	23.8	21.7	20.5	20.6	17.7	20.8	20.1	21.6	22.5	20.4	19.6	16.1	19.3	17.8	18.7	17.1	16.8
13. Tetraodon IL-4/13B	50.0	46.9	39.3	37.3	35.4	37.9	42.3	38.1	41.4	43.0	38.8	41.7		39.8	25.4	19.1	24.0	22.1	21.6	21.8	16.7	18.8	17.9	18.0	24.6	17.9	19.0	17.7	17.0	19.7	19.4	17.8	17.8	22.0	18.1	18.2	20.5	14.8	21.7	15.1	13.9	17.7	18.8	21.1	19.1	17.4	16.0	19.3
14. Fugu IL-4/13B	44.4	42.1	42.7	42.0	45.6	41.2	46.5	43.9	42.1	43.7	37.4	38.1	58.8		23.4	18.8	22.4	21.9	22.5	19.9	18.2	14.2	15.6	18.2	19.7	23.2	20.5	19.4	14.3	20.6	17.5	21.8	18.9	22.9	22.0	21.1	18.8	24.7	19.0	13.9	15.1	20.0	21.1	21.7	20.7	17.5	18.4	21.1
15. Stickleback IL-4/13B	42.3	39.3	36.7	39.3	40.8	41.2	45.1	43.9	34.3	41.5	41.0	38.8	49.6	42.1		20.9	25.2	25.9	18.8	23.1	20.1	22.0	21.7	16.7	17.9	21.9	22.4	20.0	20.5	18.0	17.0	18.9	18.1	19.5	17.5	17.5	17.6	17.2	18.4	19.3	19.7	18.4	19.0	17.3	18.5	15.5	17.4	16.8
16. Zebrafish IL-4/13A	49.0	49.0	42.7	40.8	43.3	42.7	49.7	38.2	43.9	42.7	44.6	43.3	36.9	34.4	38.2		45.9	23.4	24.1	24.4	18.9	16.3	19.8	19.5	18.4	19.7	19.0	22.3	18.9	24.4	23.2	19.0	23.4	21.2	17.1	19.7	15.9	17.5	16.1	20.2	21.7	18.7	19.4	18.4	19.9	20.6	21.5	19.6
17. Carp IL-4/13A	49.3	47.6	43.3	43.3	46.3	47.1	47.9	40.3	47.1	46.7	46.0	44.6	43.7	42.2	47.4	58.6		27.7	21.7	24.8	21.8	21.3	23.2	22.4	21.1	26.2	24.7	26.9	19.9	25.9	22.8	23.1	20.3	22.9	19.5	22.6	17.6	19.6	20.7	22.0	23.0	24.0	18.8	18.2	16.5	13.5	15.6	14.7
18. Zebrafish IL-4/13B	34.5	35.2	34.0	36.7	40.1	39.2	40.1	43.2	40.0	38.5	38.8	35.3	40.5	41.5	43.6	36.9	41.5		45.3	26.6	19.5	22.4	21.4	25.5	23.6	19.6	20.5	22.2	24.0	30.3	19.4	18.2	19.4	25.0	21.7	21.2	12.2	17.6	16.4	20.1	21.7	18.5	18.0	20.5	19.1	16.4	18.7	18.7
19. Carp IL-4/13B	43.7	37.2	36.7	36.7	41.5	39.9	47.2	43.2	35.7	37.0	39.6	43.2	42.0	39.2	44.4	38.9	42.2	60.6		24.3	20.4	16.4	21.2	22.7	23.8	20.9	19.3	24.2	22.0	24.5	16.7	15.0	14.7	20.5	19.6	19.6	18.2	18.5	14.9	20.5	21.9	21.7	21.2	21.8	18.2	18.8	16.4	15.2
20. Shark IL-4/13A	43.7	38.6	36.7	33.3	37.4	34.6	38.0	41.0	42.9	41.5	35.3	41.0	45.0	41.2	42.1	37.6	41.5	47.3	44.3		32.0	31.9	23.0	23.9	25.9	26.6	25.7	27.1	25.4	28.5	23.9	21.6	22.5	17.2	18.8	16.3	19.9	18.0	16.1	17.0	22.5	19.0	18.0	16.9	16.4	16.4	20.1	16.3
21. Shark IL-4/13B	41.1	41.8	42.0	41.3	37.4	41.2	41.1	40.4	39.7	41.1	43.2	41.8	39.0	37.0	43.2	39.5	41.8	39.0	40.4	53.4		28.4	21.9	22.1	25.2	25.6	28.2	23.8	20.1	21.9	22.4	20.5	21.8	22.9	18.0	21.0	21.4	22.5	19.2	20.1	23.8	21.3	20.1	21.4	18.0	17.5	17.7	18.9
22. Alligator IL-4 /13	38.7	35.9	32.7	38.0	30.6	35.9	46.5	39.6	36.4	41.7	40.3	39.6	36.0	34.5	40.3	35.0	43.2	43.2	39.6	49.6	46.6		21.9	28.4	29.7	25.2	26.4	32.9	26.3	31.7	16.1	18.8	18.8	21.9	20.6	18.3	16.4	23.9	18.8	20.9	17.9	20.5	19.7	18.6	16.4	18.0	17.0	15.6
23. Xenopus IL-4 /13	40.8	35.9	37.3	33.3	42.2	33.3	46.5	40.3	39.3	46.3	40.3	41.0	39.7	37.5	39.7	38.9	47.8	41.2	39.7	44.9	46.6	48.9		24.3	18.4	21.0	21.1	23.5	21.1	25.3	25.8	20.7	25.9	20.6	16.8	20.9	21.7	19.2	19.6	18.6	13.9	17.1	18.7	22.5	23.5	18.0	16.8	18.3
24. Chicken IL-4	39.4	40.7	37.3	40.0	32.7	30.1	35.2	38.8	42.1	33.1	36.0	39.6	39.7	39.7	39.7	36.9	42.6	42.6	42.6	43.4	39.7	50.4	44.9		75.0	25.7	27.0	26.1	22.7	25.3	24.2	21.8	18.5	21.2	18.4	19.6	19.3	21.2	20.4	19.8	19.8	16.8	17.4	17.3	17.2	16.7	19.5	20.7
25. Duck IL-4	40.1	37.9	34.0	38.0	33.3	34.6	40.8	43.2	41.4	43.0	36.7	39.6	41.8	38.1	39.6	38.2	43.0	37.3	44.8	50.0	44.5	57.6	39.0	80.1		20.4	22.4	24.5	26.5	28.0	24.5	26.4	17.9	18.8	22.3	18.8	25.2	24.0	25.2	21.1	22.1	20.1	26.4	19.9	19.1	16.9	20.0	20.7
26. Chicken IL-13	34.5	37.2	36.0	39.3	38.8	36.6	43.0	41.0	37.9	38.4	40.3	36.7	37.0	35.5	39.9	37.6	42.8	37.7	39.1	44.9	45.2	46.8	44.9	48.6	40.6		79.9	22.6	22.5	20.1	18.6	25.5	21.7	17.5	23.0	19.3	17.6	20.1	19.5	17.2	19.1	14.8	19.0	16.7	17.4	19.2	14.5	19.5
27. Duck IL-13	39.4	36.6	38.0	40.0	40.8	37.3	42.3	43.9	38.6	39.6	37.4	36.7	37.4	38.8	41.0	40.1	42.4	38.1	38.8	45.3	50.0	47.5	44.6	43.9	41.0	88.5		22.5	23.0	17.4	20.1	24.7	21.2	16.7	17.0	18.6	18.4	21.7	20.8	18.4	14.7	17.3	15.9	17.1	15.5	19.8	17.2	18.6
28. Human IL-4	39.9	40.5	33.3	35.9	34.0	36.6	44.4	39.9	35.3	39.2	42.5	39.9	33.3	37.9	30.1	41.4	41.8	37.3	39.2	45.8	44.4	47.7	39.2	43.8	43.8	39.2	40.5		40.3	56.8	23.4	20.1	22.5	22.1	18.4	20.5	24.3	22.6	22.1	17.2	22.8	17.2	22.5	21.4	21.5	18.6	17.7	17.5
29. Mouse IL-4	35.2	38.6	41.3	38.0	36.7	37.3	36.6	37.9	38.6	41.4	40.7	41.4	37.9	39.3	38.6	33.8	39.3	44.3	40.0	45.7	39.7	46.4	38.6	47.9	46.4	39.3	40.7	56.9		42.5	20.3	19.5	20.7	21.5	16.0	16.6	17.8	24.1	15.8	20.5	22.6	22.4	19.2	22.5	21.1	21.6	19.9	25.0
30. Cow IL-4	37.3	37.9	32.7	33.3	31.3	34.6	45.8	41.7	42.1	39.3	43.2	43.2	38.5	35.6	33.3	35.7	41.5	50.4	41.5	45.2	41.1	46.8	45.6	45.6	45.2	39.1	38.1	64.7	55.0		22.3	21.6	21.9	20.3	18.9	18.5	23.1	22.1	21.7	18.5	24.4	20.9	18.4	21.3	22.1	19.8	17.4	22.9
31. Human IL-13	37.7	37.0	32.0	34.0	36.1	41.8	41.1	34.9	39.7	37.7	34.2	33.6	39.0	39.7	37.7	43.3	43.8	30.8	34.9	47.3	42.5	37.0	40.4	40.4	44.5	37.7	38.4	38.6	39.0	40.4		52.7	64.4	14.7	16.5	16.6	21.0	18.3	19.1	20.8	17.8	19.0	18.3	18.8	17.9	23.5	20.1	18.6
32. Mouse IL-13	35.9	35.9	32.7	36.7	38.8	37.9	36.6	40.3	36.4	37.0	39.6	35.3	36.6	42.0	36.1	37.6	40.7	32.1	38.9	47.3	39.0	34.5	46.3	42.6	44.0	44.9	43.9	36.6	35.0	38.5	65.8		53.7	14.9	17.9	14.3	20.5	20.5	18.5	18.2	15.1	21.1	15.8	16.7	15.0	16.0	19.9	19.9
33. Cow IL-13	38.0	41.4	33.3	38.0	36.7	37.9	41.5	39.6	43.6	39.3	40.3	40.3	38.6	39.4	39.8	42.7	40.7	40.2	34.1	48.5	40.4	38.8	44.9	39.7	44.0	43.5	43.2	37.3	37.9	40.0	74.7	72.7		14.1	16.9	16.8	21.6	19.4	20.0	18.8	19.7	15.3	14.6	19.1	20.5	20.4	17.8	14.4
34. Human IL-2	38.6	36.6	36.6	35.3	37.3	39.2	43.1	38.6	37.9	39.9	39.9	37.3	32.7	37.9	40.5	40.1	40.5	35.9	34.6	34.0	41.8	35.3	39.9	34.6	34.6	35.3	41.2	35.9	35.3	34.0	34.6	32.0	33.3		65.8	65.2	16.7	19.3	19.8	20.0	21.9	21.1	19.8	17.0	16.6	20.6	23.2	21.0
35. Rat IL-2	42.6	40.6	38.1	35.5	38.1	43.2	41.3	35.5	40.0	31.6	39.4	40.6	31.6	36.1	42.6	36.9	38.1	40.0	37.4	38.7	40.6	40.0	41.3	38.7	37.4	40.6	34.2	34.2	37.4	36.1	38.1	34.8	34.8	78.1		51.3	18.1	17.1	18.5	21.9	21.5	23.6	19.2	19.3	18.3	19.2	20.1	19.2
36. Cow IL-2	38.1	33.5	40.0	38.7	36.8	38.1	41.9	37.4	37.4	39.4	39.4	32.9	34.2	35.5	41.3	40.1	40.6	36.1	36.1	35.5	42.6	40.0	38.1	38.1	38.1	37.4	38.7	34.2	36.1	35.5	39.4	34.2	37.4	77.4	68.4		18.3	21.0	17.0	18.7	21.8	19.5	17.0	17.2	12.6	19.8	20.8	19.1
37. Human IL-9	39.6	37.2	32.0	35.3	39.5	39.2	34.7	36.1	39.6	38.2	40.3	41.0	38.9	38.9	36.1	33.8	38.2	33.3	38.2	41.0	36.3	34.7	41.7	38.9	45.1	32.6	32.6	39.9	36.8	38.9	43.8	39.6	42.4	32.7	33.5	34.2		56.3	59.0	21.5	21.5	20.2	18.1	17.4	20.9	17.4	18.4	18.9
38. Mouse IL-9	41.0	37.9	32.7	37.3	37.4	34.0	41.0	35.4	38.2	41.0	40.3	37.5	31.3	41.0	38.2	35.7	34.7	35.4	33.3	37.5	39.0	40.3	37.5	40.3	39.6	36.1	35.4	39.9	42.4	40.3	42.5	38.2	38.9	36.6	30.3	37.4	70.8		47.2	20.1	19.8	19.8	17.6	18.4	19.5	18.4	15.4	19.5
39. Cow IL-9	37.5	35.2	30.7	34.0	34.0	35.3	41.7	30.6	35.4	43.1	37.5	41.7	34.7	34.7	34.7	33.8	37.5	29.9	37.5	34.0	32.9	38.9	33.3	40.3	46.5	32.6	38.2	39.2	38.9	37.5	40.4	38.9	38.2	39.9	34.8	36.1	75.0	68.8		18.4	20.9	18.1	20.2	21.0	22.3	16.9	17.9	16.5
40. Human IL-21	40.0	40.6	35.5	38.7	37.4	46.5	44.5	36.1	40.6	36.1	39.4	40.6	32.3	33.5	38.7	40.1	44.5	39.4	34.8	36.1	40.0	39.4	41.3	35.5	40.6	34.2	33.5	38.7	42.6	38.1	38.1	34.2	34.2	40.0	45.2	36.1	36.1	36.1	38.1		59.4	76.8	20.3	15.6	19.8	21.2	16.3	20.0
41. Mouse IL-21	39.7	34.2	36.0	36.7	35.4	39.9	40.4	36.3	37.0	41.1	37.0	39.7	33.6	30.8	41.8	37.6	43.8	37.0	37.7	37.0	43.8	40.4	32.2	39.0	38.4	37.0	32.2	37.3	43.8	39.7	34.9	30.1	39.7	41.8	42.6	43.9	37.7	36.3	42.5	74.2		67.8	19.2	20.0	19.2	18.1	22.3	20.1
42. Cow IL-21	41.8	41.1	38.0	38.7	37.4	42.5	46.6	34.9	38.4	39.7	38.4	39.7	36.3	35.6	44.5	36.3	43.8	37.7	38.4	39.7	43.2	37.7	39.0	38.4	43.2	35.6	37.0	39.2	44.5	41.1	37.7	33.6	34.9	40.5	39.4	36.1	42.5	39.7	39.0	84.5	80.1		21.6	18.2	19.5	20.7	17.9	20.1
43. Human IL-7	32.2	33.9	33.9	33.3	35.6	35.0	39.0	35.0	34.5	40.1	31.6	30.5	29.4	33.3	33.3	38.4	34.5	32.2	33.9	33.3	43.5	35.0	36.7	36.2	37.3	31.1	29.9	34.5	36.7	35.6	35.6	32.2	32.8	35.0	36.2	35.6	31.1	31.1	35.0	39.0	39.0	40.7		60.5	75.1	20.2	17.4	17.6
44. Mouse IL-7	35.7	37.7	36.4	39.0	39.0	36.4	37.0	35.7	38.3	36.4	31.2	35.1	34.4	34.4	36.4	42.0	35.7	37.0	38.3	33.8	40.9	39.6	39.6	40.3	34.4	31.8	33.8	37.7	39.6	37.0	38.3	30.5	36.4	38.3	37.4	35.5	35.7	30.5	33.1	38.7	40.9	42.2	72.3		64.8	17.6	21.5	20.5
45. Cow IL-7	34.7	36.4	36.9	32.4	35.8	36.9	36.4	33.5	39.2	33.5	34.1	30.7	30.1	34.7	30.7	36.4	33.0	31.8	34.1	31.3	36.4	29.5	37.5	35.2	33.5	31.8	34.1	33.0	36.4	35.8	35.2	27.3	31.8	34.7	31.3	34.1	34.1	31.3	35.8	36.9	38.6	43.2	82.5	75.6		21.4	19.5	19.9
46. Human IL-15	35.2	35.2	36.4	34.6	38.3	34.6	36.4	31.5	34.6	33.3	36.4	32.1	34.0	34.0	36.4	42.0	35.8	35.8	35.2	33.3	35.8	37.0	34.6	34.0	34.0	34.0	37.7	37.7	42.6	35.8	44.4	29.0	38.9	41.4	40.1	40.1	32.7	35.8	38.9	42.6	39.5	40.1	36.2	37.7	38.6		72.8	78.4
47. Mouse IL-15	34.6	31.5	34.0	32.7	35.8	35.2	34.6	32.7	37.7	38.3	37.7	31.5	27.2	34.0	32.7	39.5	35.8	34.6	34.0	31.5	37.0	34.0	34.0	39.5	36.4	34.0	34.6	34.6	38.3	36.4	37.0	37.0	34.0	38.9	38.9	44.4	33.3	30.9	36.4	40.1	41.4	40.1	35.0	39.5	36.9	84.6		72.2
48. Cow IL-15	35.8	37.0	36.4	32.1	35.8	33.3	38.3	35.8	34.0	36.4	39.5	32.7	30.9	35.2	32.7	42.6	35.2	37.0	33.3	34.6	37.0	35.2	32.7	40.7	36.4	32.1	36.4	37.0	39.5	37.0	37.0	34.0	35.8	43.2	46.9	38.3	33.3	38.3	37.0	38.9	37.0	40.1	33.9	35.2	35.2	88.9	82.1	

Table S2 Primers used for expression analysis by real-time PCR

Gene	Forward (5' to 3')	Reverse (5' to 3')	Acc. No.
House-keeping gene			
EF-1α	CAAGGATATCCGTCGTGGCA	ACAGCGAAACGACCAAGAGG	AF498320
Acute phase protein and antimicrobial peptide genes			
Serum amyloid A	GGTGAAGCTGCTCAAGGTGCTAAAG	GCCATTACTGATGACTGTTGCTGC	AM422447
SAP1	GCTGTTATGGTGACCTTCAAGATCTCTC	GCGTTTGTACAACAACAAATCATTGTC	X99385
SAP2	GGTTGTTATGCTGAACATCAAGATCTCTC	CCACCCTTTGATTGCATACACAGATT	EZ763346
CATH1	ACCAGCTCCAAGTCAAGACTTTGAA	TGTCCGAATCTTCTGCTGCAA	AY594646
CATH2	ACATGGAGGCAGAAGTTCAGAAGA	GAGCCAAACCCAGGACGAGA	AY542963
Hepcidin	GCTGTTCCTTTCTCCGAGGTGC	GTGACAGCAGTTGCAGCACCA	CA369786
Cytokines			
IL-1β1	CCTGGAGCATCATGGCGTG	GCTGGAGAGTGCTGTGGAAGAACATATAG	AJ278242
IL-4/13A	ACCACCACAAAGTGCAAGGAGTTCT	CACCTGGTCTTGGCTCTTCACAAC	FN820501
IL-4/13B1	GAGATTCATCTACTGCAGAGGATCATGA	GCAGTTGGAAGGGTGAAGCTTATTGTA	HG794522
IL-4/13B2	GAGACTCATCTATTGCGTATGATCATCG	TGCAGTTGGTTGGATGAAACTTATTGTA	HG794523
IL-8	AGAGACACTGAGATCATTGCCAC	CCCTCTTCATTTGTTGTTGGC	AJ310565
IL-10a	GGATTCTACACCACTTGAAGAGCCC	GTCGTTGTTGTTCTGTGTTCTGTTGT	AB118099
IL-10b	GGGATTCTAGACCACATCAAGAGTCC	GATGGGAGATTTAAAGTTGTGTGTTCC	FR691804
IFNγ1	CAAACTGAAAGTCCACTATAAGATCTCCA	TCCTGAATTTTCCCCTTGACATATTT	AJ616215
IFNγ2	CAAACTGAAAGTCCACTATAAGATCTCCA	GGTCCAGCCTCTCCCTCAC	FM864345
TNFα1	TGTGTGGGGTCCTCTTAATAGCAGGTC	CCTCAATTTCATCCTGCATCGTTGA	AJ277604
TNFα2	CTGTGTGGCGTTCTCTTAATAGCAGCTT	CATTCCGTCCTGCATCGTTGC	AJ401377
TGF-β1A	CTCACATTTTACTGATGTCACTTCCTGT	GGACAACTGCTCCACCTTGTG	OMY7836
TGF-β1B	CATGTCCATCCCCCAGAACT	GGACAACTGTTCCACCTTGTGTT	FN822750
LECT2	CCTCAGTGGAGAAGGTCTGTGCT	TCCATTAGAAGAACTTGGTGGGGT	CA367917
M17	GTGGACCTCTTAAAAACATACAAGCTCAG	GGATGGTGGCTGTAAGTCTGTCTG	FM866399
Cytokine receptors			
IL-4Rα1	CTGAATACAGCATGACGTTGAAACA	GGGGTTTTGGGTTTTATGGTGT	AJ634731
IL-4Rα2	GTGCTGAATACAACATGACATTGGAAT	GGGTTTTGGGTTTTATGCTGC	FN824519
IL-13Rα1a	CTGGACTGAGGAGGAAATATATGGTAAAG	TGGGATGTAGAGGTTCTCATTAATGC	FN824520
IL-13Rα1b	TGCAGTTGAGAAGGAGTTGAAGAACTT	TGCATGCTGTTTTGTGGGATTC	FN824521
IL-13Rα2a	ATGGATGGTGCCCGGAGGAA	CCCTCTCTGGCTTTCCACTTTCTCC	NM_001124308
IL-13Rα2b	ATGGATGGAGCCCACTGCTT	CCCTCTCTGCCTTTACACTTTGTCC	FN824522
IFNγR1	AAGCATGGGGATTCTGGTCCT	CCGATATGTGACAGTATGGAAGC	EU244876
IFNγR2	CAGACAGGCCAGGGATAAAGT	TACCATGTGGACCATCAGAAG	EU244877
IL-6Rα	CAACACCTGGACAGCCCCTG	CACAGGAAGCAACCACCCACA	FN824530
GP130	GCGTCCTGTTTGTCATAGTGCTAACTG	CCTTGGGACTGACAGCTTTGGT	FN824531
MCSFR1	CAGACTTTGCACCTCCAGAGATGTATAC	GTTCTGGTGCTGCTGGGACG	AJ417832
MCSFR2	GACTTTGCCCCTCCAGAGATATACAC	CACAATCCTCACTAATCTTAGCTTGGC	AB091826
SOCS			
SOCS1	GATTAATACCGCTGGGATTCTGTG	CTCTCCCATCGCTACACAGTTCC	AM748721
SOCS2	GGATCCACTCGCAAATAGGACGATAC	GGATTCGGGTGAGTGGCAGGT	AM748722
SOCS3	CACAGAGAAACCGTTAAAAGGACTATCC	AAGGGGCTGCTGCTCATGAC	AM748723

Table S2, continued

Gene	Forward (5' to 3')	Reverse (5' to 3')	Acc. No.
T cell markers			
CD4-1	GTGTGGAGGTGCTACAGGTTTTTTC	ATCGTCACCCGCTGTCTGTG	AY973028
CD4-2A	CGACATTGTCACAGTCAAGGTCC	CCTCATTTGGCAACAAACTTCTCAC	AY973029
CD4-2B	CGACATTGTCACAGTCAAGGTCC	GGCTTAGCAGTCAACAAACTTTTCAC	AY899932
CD8α	CCAAGTCGTGCAAAGTGGGAA	CTTGGCTGTCTTTTGTAATGATGTGG	AF178053
CD8β	GAACTATCAAACCCCAGAAGGCTGTG	GACACTTTTTGGGTAGTCGGCTGAA	AY563420
CD28	CTTGGTCCTGGTTGAAGCATTTC	CGAAGACCGTGACAACCAGAC	AY789435
CTLA4	ACCCACAACAACACAGCCTTCC	GGTAAAGCACCCTGGTTCCTCTGTTAT	CA044588
T-bet	GGTAACATGCCAGGGAACAGGA	TGGTCTATTTTTAGCTGGGTGATGTCTG	FM863825
GATA3	CCAAAAACAAGGTCATGTTCAGAAGG	TGGTGAGAGGTCGGTTGATATTGTG	FM863826
FoxP3a	CCCAGAACCGAGGTGGAGTGT	TGACGGACAGCGTTCTTCCA	FM883710
FoxP3b	TCCTGCCCCAGTACTCATCCC	TGACGGACAGCGTTCTTCCA	FM883711
RORγ	ACAGACCTTCAAAGCTCTTGGTTGTG	GGGAAGCTTGGACACCATCTTTG	FM883712-3
Macrophage/dendritic cell markers			
CD83	GTGAGGTGGTACAAGCTGGGTG	GCTGCCAGGAGACACTTGTACCT	AY263797
CLEC4	CACCACTGACCACAGCGAATTG	GAACATTTCTCATCATTCCACCACC	FN667662
CD209L1	TGTGCTGATTGGTCTATTGGCATC	ACACTCCCTACACTTCCTTACTGTCTTTG	AY593994
CD209L2	CACCTTAGCATCCTGCACAGCAA	CGAGCTGTACGGTTGCCAGAAGTTAT	FJ607865
LAMP3	ACATGAAAAGCTGTTCCCAACTGC	AGCCCAGCACTCAACCTCCTC	NM_001281412
CD80/86	CAGGAACACACTGTCTGCAGGC	CTGCTCCCTTTCCTCCTTGATTACTTC	EU927451
MHC I	TGCATTGAKTGGCTDAAGAAGTAT	TCCAAATGACGACCCCAACAAC	**
MHC IIβ	TCAGATTCAACAGCACTGTGGGGAG	CTTCTTCTTGTAGTAGATCAGTCCTGCT	**
B cell markers			
IgM H, secreted	TACAAGAGGGAGACCGGAGGAGT	CTTCCTGATTGAATCTGGCTAGTGGT	X65261
IgM H, membrane	CCTACAAGAGGGAGACCGATTGTC	GTCTTCATTTCACCTTGATGGCAGT	OMU04616
IgD H, secreted	TGAACATATCCAAACCAGGTGTCTG	GTCCTGAAGTCATCATTTTGTCTTGA	JQ003979
IgD H, membrane	TGAACATATCCAAACCAGAGCTCC	GTCCTGAAGTCATCATTTTGTCTTGA	AY870260
IgT H, secreted	CATCAGCTTCACCAAAGGAAGTGA	TCACTTGTCTTCACATGAGTTACCCGT	AY870268
IgT H, membrane	TCGAAGTCCACGGCGAACA	GTGTTCTTCACCGCTTCATCTTGAA	AY870264

Note:

** Primers for the polymorphic trout MHC genes were designed from the Immuno Polymorphism Database (IPD)-MHC FISH database (http://www.ebi.ac.uk/ipd/mhc/fish/).
